# Intriguing physicochemical properties and impact of co-dopants on N-doped graphene oxide based ZnS nanowires for photocatalytic application

**DOI:** 10.1038/s41598-023-33453-z

**Published:** 2023-05-10

**Authors:** D. V. Dake, N. D. Raskar, V. A. Mane, R. B. Sonpir, E. Stathatos, M. Vasundhara, R. Meena, K. Asokan, B. N. Dole

**Affiliations:** 1 Advanced Materials Research Laboratory, Department of Physics, Dr. Babasaheb Ambedkar Marathwada University, Aurangabad, 431004 M.S. India; 2 Electrical and Computer Engineering Department, Nanotechnology and Advanced Materials Laboratory, University of the Peloponnese, 26334 Patras, Greece; 3 Polymer and Functional Department, CSIR-Indian Institution of Chemical Technology, Tarnaka, Hyderabad, Telangana 500007 India; 4 Materials Science, Inter-University Accelerator Centre, Aruna Asaf Ali Marg, New Delhi, 110 067 India; 5 Department of Physics and Centre for Interdisciplinary Research, University of Petroleum and Energy Studies (UPES), Dehradun, Uttarakhand 248007 India

**Keywords:** Materials science, Nanoscience and technology

## Abstract

Superparamagnetic N-doped graphene oxide (GO)- with ZnS nanowires was synthesized by a one-step hydrothermal method by doping dilute amounts of Ga, Cr, In, and Al ions for water treatment and biomedical applications. In these experiments, to enhance their properties, 2% of Ga^3+^, In^3+^, and or Al^3+^ were codoped along with 2% Cr ions in these ZnS nanowires. The nanocomposite with the composition, In_0.02_Cr_0.02_Zn_0.96_S, has better photocatalytic efficiency than other co-doped nanocomposites. The In (metalloids) and Cr (transition metal ion) are the best combinations to increase the magnetic properties which are beneficial for photocatalytic activity. Synthesized nanocomposite materials were characterized by several techniques such as X-ray diffraction, Field emission-scanning electron microscope (FESEM) with EDAX, vibrating sample magnetometer (VSM), UV–Vis, X-ray photoelectron spectroscopy (XPS), and fluorescence spectroscopy. The correlation of intriguing magnetic properties with their photocatalytic properties is also discussed. XPS was employed for the detection of surface defects, phase transformation, and the nature of chemical components present in the nanocomposites. The Frankel and substitutional defects have a direct impact on photocatalytic activity that was determined from the fluorescence (FL) spectroscopy. FL and XPS reveal that the Cr and In codoped composite has a higher percentage of defects hence its photocatalytic efficiency reaches 94.21%.

## Introduction

Over the past decade, graphene oxide (GO)^1–5^ and co-doping of 3d metals or rare earth metals, non-metals, and metalloids have received enormous attention due to their key role in the enhancement of those physical properties which are applicable in industries such as tensile strength, mobility, conductivity, bandgap, flexibility, thermal and chemical stability, young’s modulus, optical transparency, high electroconductivity, desirable to production, etc.^6,7^. Zinc sulfide (ZnS) is an II-VI semiconductor photocatalyst and it has been extensively studied because it appears in several morphologies at the nanoscale, exhibits excellent physical properties, and also has unique photocatalytic properties. However, there are few reports available in the literature on co-substituting with Cr^3+^ ions in ZnS^8,9^. Cr (VI) is a heavy metal and also highly toxic, whereas Cr (III) is nontoxic and useful for human beings. Therefore, in addition to that, graphene oxide nanosheets were used to tune the bandgap of pure ZnS from the UV region to the visible region to utilize more solar energy^10^. TiO_2_ is the most commonly used nanostructured material^11^. However, in environmental applications, pure ZnS has greater theoretical efficiency in the generation of photo carriers than TiO_2_^12^. Besides, nanocrystals of co-substituted semiconductors have a better ability to impart new technological applications and are beneficial for diverse properties compared to the pure nanocrystal material. It was explicitly corroborated that co-substituting is a more effective tool than single metal/nonmetal/metalloids doping into host nanocrystals which are used to improve the properties of the photocatalyst^13^. In principle, the morphology, structural defects, and surface defects of metal sulfides and graphene metal sulfide nanocomposites play a vital role in controlling transport properties, chemical and thermal stability, and excitonic movements. Suvanka Dutta et al.^14^ and Gajendiran et al.^15^ reported that the properties of mixed morphology of prepared samples are most important rather than their homogeneity and single-crystalline samples.

From the data presented in Table 1S (EIS), it is evident that co-doped ZnS has better results than single metal-doped ZnS nanomaterials. The present study focuses on the effects of co-doping of 2% Ga^3+^/In^3+^/Al^3+^ ions along with 2% Cr^3+^ ions on the GO-adorned ZnS nanowires and their photocatalytic properties. After the co-doping of metaloids into ZnS lattice, more defects were formed lading to higher photocatalytic dye degradation efficiency, the direct impact of defects on the efficiency of photcatalytic activity is discussed in detail.

## Experimental

### Material synthesis

#### Synthesis of N-doped graphene oxide ultrathin nanosheets from coal stone

In this work, we have used GO nanosheets for the application of photocatalytic activity. The synthesis procedure was followed as per the previous publication^16^ without further annealing process. GO has been successfully prepared from coal stone. Thioura was added to the aforementioned solution in 1:2 ratio after dissolving the GO powder in DI water. A 30-min ultrasonic treatment was given to the solution. The sample was put into a stainless steel autoclave and sealed after being sonicated. For 3 h, a sealed autoclave was kept in a furnace at 90 °C. The autoclave was cooled to room temperature, the solution was filtered, it was washed several times in DI water, and it was dried at room temperature. Then, the final product was N-doped GO which was dried at 60 °C^17–19^.

#### Synthesis of graphene oxide decorated Cr substituted ZnS nanowires

Graphene oxide adorned ZnS nanowires co-substituted 2% Ga^3+^/In^3+^/Al^3+^ and 2% Cr^3+^ ions prepared using the hydrothermal technique. Homogeneous solutions of Zn, Cr, Ga, In, and Al salt compounds, with S^2−^ as a precipitating anion formed by the decomposition of Thiourea. GZ (GO:ZnS) nanowires were prepared using 1 M of zinc acetate dihydrate, 3 M thiourea, and 0.2 g of graphene oxide were separately dissolved in 15 ml of DI water and 15 ml of ethylenediamine (1:1) in each beaker. The Zinc acetate dihydrate solution was placed on the magnetic stirrer for constant stirring to a 100% clear solution. The same procedure was applied to each solution. After the homogeneous solution was formed, the first thiourea was added into the zinc acetate solution and stirred for 20 min, and then the graphene oxide solution was added to the above solution. The solution was constantly stirred for 2 h while maintaining a pH of 10 by adding ammonia dropwise. The homogeneous solution was transferred to a Teflon-lined stainless steel autoclave and sealed carefully. The sealed autoclave was held at 190 °C in a muffle furnace for 12 h. The system was cooled to room temperature and the formed precipitate was filtered and washed several times with DI water and ethanol. The washed precipitate was dried at room temperature before being calcined overnight at 60 °C. The zinc acetate dihydrate 0.96 M, 0.02 M of gallium chloride /indium chloride and aluminum chloride 0.02 M of chromium chloride, and 3 M thiourea were used for the preparation of nanocomposites. The aforementioned procedure was followed for Graphene oxide decorated ZnS nanowires substituted with 2% Ga^3+^/In^3+^/Al^3+^ with 2% Cr^3+^. Graphene oxide (GO) adorned ZnS nanowires are named GZ, 4% Cr doped GZ as GZ:Cr, and co-substituted 2% Ga^3+^/In^3+^/Al^3+^ and 2% Cr^3+^ ions in GZ as GZ:Cr-Ga, GZ:Cr-In and GZ:Cr-Al respectively.

### Ethical approval

No animals were used or harmed in the research or experimentation for this manuscript.

## Result and discussions

### Crystallographic study

**Figure 1:**
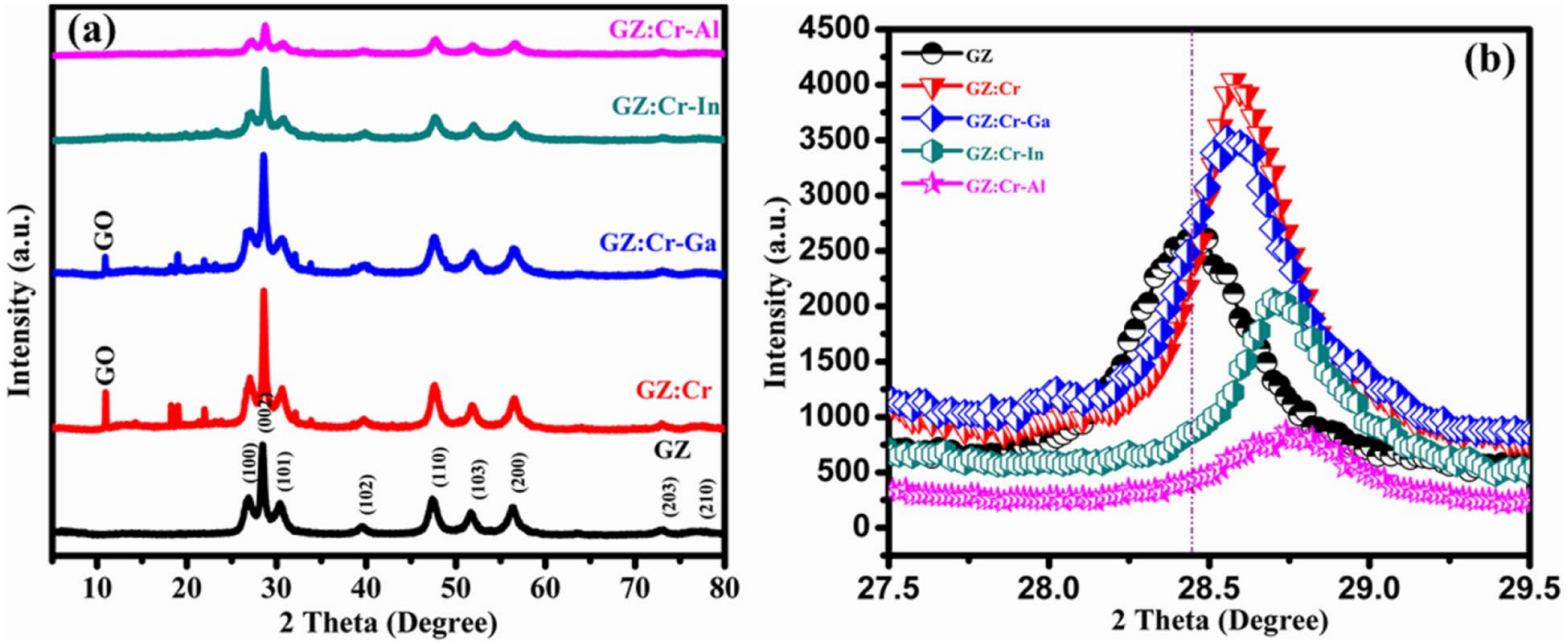
XRD plots of (**a**) GZ, GZ:Cr, GZ:Cr-Ga, GZ:Cr-In, and GZ:Cr-Al Nanowires, and (**b**) enlarged view between the angle 27.5° and 29.5°.

Crystallographic parameters such as lattice parameters, crystallite size, volume, dislocation density, microstrain, stacking faults, ‘u’ parameter, bond length, and c/a ratio were obtained using the XRD technique and are depicted in Table 1. The Wurtzite crystal structure of as-prepared sample nanocomposites was verified by the XRD technique which is depicted in Fig. 1 and well-matched with JCPDS 36-1450. The peaks of GZ nanocomposites are located at 26° (100), 28° (002), 30° (101), 39° (102), 47° (110), 51° (103), and 56° (200), and the graphene oxide peak is noticed for GZ:Cr-Ga and GZ:Cr at 10°, but notably, the GO peak is not observed in other nanocomposites due to low crystallinity or low diffraction intensity of GO. The observation that the Graphene Oxide (GO) peak is absent in XRD spectra of GZ, GZ:Cr-In, and GZ:Cr-Al nanocomposites may be due to the graphene oxide is an oxidized form of graphene and has oxygen-containing functional groups on its surface. These functional groups can be reduced to form graphene, which does not have these groups. During process of synthesis of the GZ, GZ:Cr-In, and GZ:Cr-Al nanocomposites, the reduction of GO to graphene occurred, leading to the absence of the GO peak in the XRD spectra^20^. One possible reason for the absence of the GO peak in these samples could be the presence of other compounds in the nanocomposites that could mask the GO peak in the XRD spectra. For example, in GZ, the ZnS crystal structure could be dominant, and the presence of graphene could be difficult to detect. This is supported by the fact that the GO peak was observed in the XRD spectra of GZ:Cr and GZ:Cr-Ga nanocomposites, where the dopants may not have been sufficient to mask the GO peak. Overall, the absence or presence of the GO peak in XRD spectra of the nanocomposites could be due to the reduction of GO, or the masking effect of other compounds caused by the incorporation of dopant elements^21,22^. The crystallinity decreases after co-substituting the metalloid ions into the ZnS lattice. This effect suggests that the co-substituting of metalloid ions into the ZnS lattice has succeeded. Whereas dislocation density, microstrain, and stacking faults are opposite in order meaning that crystallite size increased with decreasing dislocation density, microstrain, and stacking faults.

**Table 1:** Structural parameters of the GO/(2% Ga/In/Al) – (2% Cr) co-substituted ZnS Nanowires.

Sample name	Lattice parameters (nm)		Crystallite size (nm)	Volume (Å)	Dislocation density (lines/m^2^)	Microstrain (ε)	Stacking faults (SF)	‘u’ parameter	Bond Length (Å)	c/a ratio
	a	c								
GO/Pure ZnS (GZ)	3.8085	6.271	20.88	78.786	0.0022	0.184	0.243	0.3729	2.338	1.646
GO/4% Cr: ZnS (GZ:Cr)	3.792	6.238	23.93	77.685	0.0017	0.163	0.213	0.3731	2.327	1.644
GO/2% Ga/2% Cr:ZnS (GZ:Cr-Ga)	3.793	6.242	19.47	77.779	0.0025	0.209	0.323	0.3730	2.328	1.645
GO/2% In/2% Cr:ZnS (GZ:Cr-In)	3.781	6.210	22.67	76.923	0.0019	0.174	0.2325	0.3736	2.320	1.642
GO/2% Al/2% Cr :ZnS (GZ:Cr-Al)	3.782	6.208	19.81	76.928	0.0026	0.197	0.246	0.3737	2.320	1.641

The lattice parameters a, and c for wurtzite ZnS nanowires were calculated using the following formula^23,24^.

1 $$\frac{1}{{\mathrm{d}}^{2}}=\frac{4}{3}\left[\frac{{\mathrm{h}}^{2}+\mathrm{hk}+{\mathrm{k}}^{2}}{{\mathrm{a}}^{2}}\right]+\frac{{\mathrm{l}}^{2}}{{\mathrm{c}}^{2}}$$

There is a significant difference in crystal structure after co-substituting metalloids into the ZnS lattice sites. Debye Scherrer's formula was employed to calculate the average crystallite size. Also, when a foreign element with a different ionic radius is substituted in the ZnS lattice, a change is confirmed in crystallite size^25^.

2 $$D=\frac{K\lambda }{\beta \mathrm{cos}\theta }$$

The size of the exposure volume can help to ensure that a statistically significant number of grains are included in the diffraction pattern. This indicates that the intensity of the integrated diffraction peak is proportional to the volume of the material. The volume of as-prepared samples was evaluated using the following formula^26^.

3 $$V={a}^{2}\times c\times 0.866$$

**Figure 2:**
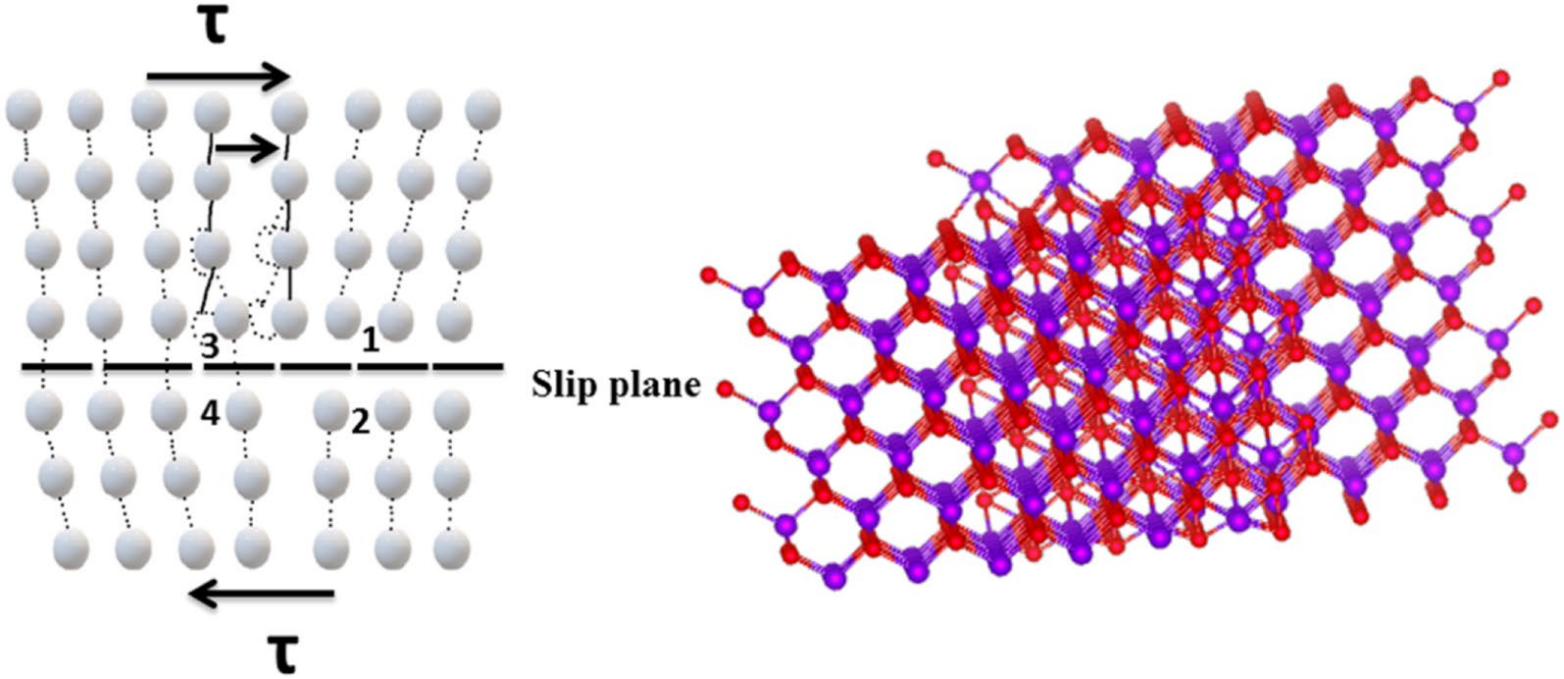
The schematic showing the dislocation in prepared samples under stress and strain.

Stacking faults can appear in a crystal as a result of plastic deformation or solidification. An ideal wurtzite crystal structure can be thought of as one of a series of stacking planes, and stacking faults are found in the samples if the order of the sequence of stacking planes is not followed precisely. Peak shifts and broadening of the peak are observed due to the presence of stacking faults, changes in the lattice parameter, and residual stress. In microstrain, the factor to consider is the general decrease in lattice characteristics when the larger ion is partially replaced by the smaller one. It causes a change in the internal structure, shape, and volume on a microscopic scale in the crystal lattice. The method of assumption of the co-integration dopants in the ZnS lattice, which is wrapped by thin graphene oxide nanosheets, as a source of extended defects brought on by the lattice mismatch (misfit dislocations), as shown in Fig. 2. The number of dislocations per unit volume of a crystalline material is measured by dislocation density. From the following equations, the crystallite characteristics of all samples, dislocation density, microstrain, and existent stacking faults were analyzed^16^.

4 $$\mathrm{Dislocation\,density }\left(\delta \right)=\frac{1}{{D}^{2}}$$

5 $$Microstrain\left( \varepsilon \right)=\frac{\beta \mathrm{cos}\theta }{4}$$

6 $$Stacking\,faults \left(SF\right)=\left[\frac{2{\pi }^{2}}{45{\left(3\mathrm{tan}\theta \right)}^\frac{1}{2}}\right]\beta $$

From the following formula, the ‘u’ parameter of wurtzite structured ZnS was calculated

7 $$\mathrm{u}=\frac{{\mathrm{a}}^{2}}{3{\mathrm{c}}^{2}}+0.25$$

Bond length is proportional to bond order: a stronger bond will be shorter if a large number of electrons participated in its creation. Bond length is inversely related to bond strength and bond dissociation energy: a stronger bond will be smaller if all other parameters are equal. The bond length (L) was scrutinized using the following formula^27^.

8 $$\mathrm{L}=\sqrt{\frac{{\mathrm{a}}^{2}}{3}+{\left(\frac{1}{2}-\mathrm{u}\right)}^{2 }{\mathrm{c}}^{2}}$$

### Optical study

#### UV–Vis reflectance spectroscopy

**Figure 3:**
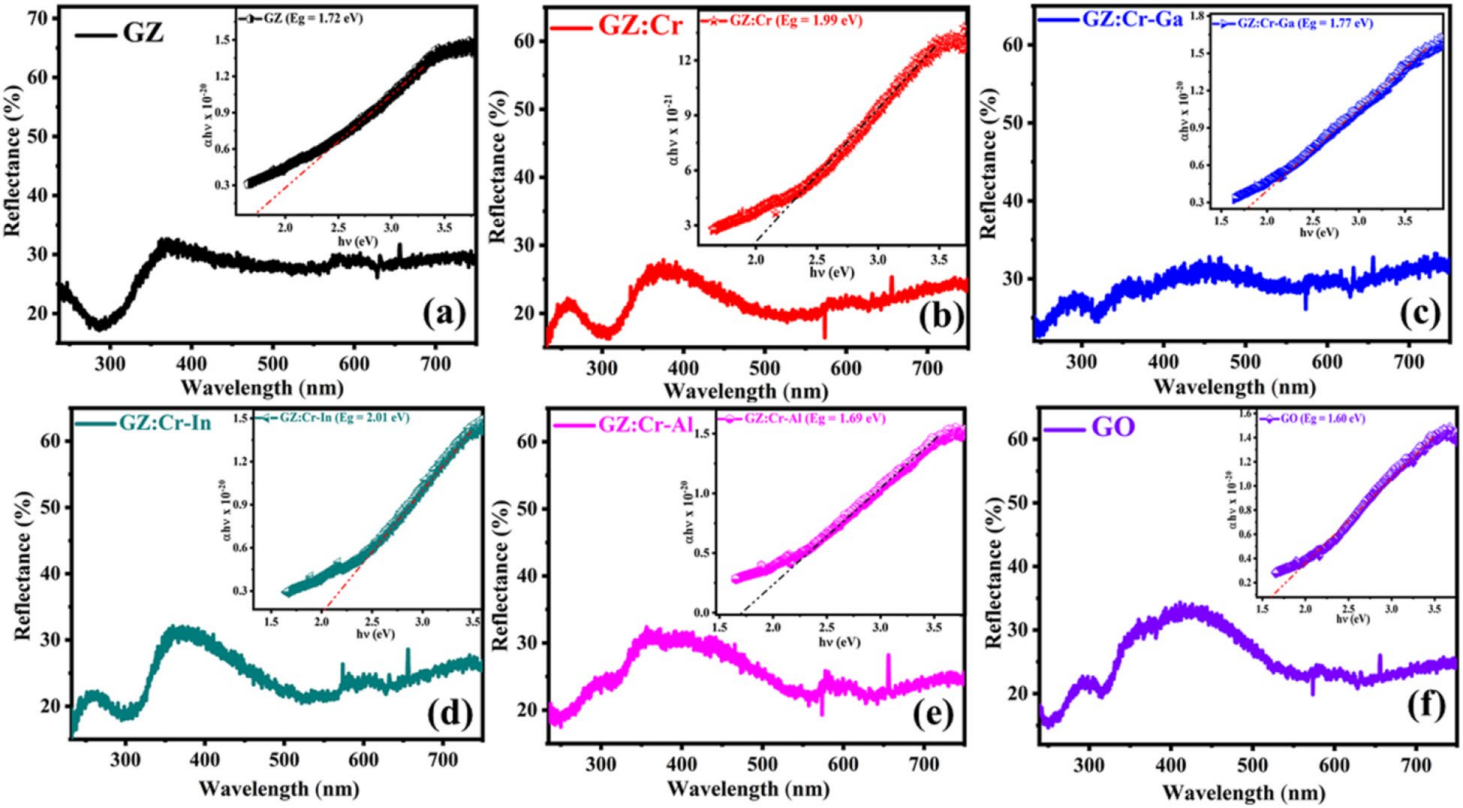
UV–Vis reflectance spectra with insets showing the bandgap plots of (**a**) GZ, (**b**) GZ:Cr, (**c**) GZ:Cr-Ga, (**d**) GZ:Cr-In, (**e**) GZ:Cr-Al, (**f**) GO.

UV–Vis reflectance spectroscopy determines the optical properties such as the bandgap of as-prepared semiconductor samples. The reflectance spectra were recorded in the range of 200 nm to 750 nm and they reflect the bands in the visible region which are depicted in Fig. 3. It was explicitly corroborated that as-synthesized samples absorb maximum solar light by reducing the bandgap in the visible region. Prepared samples have been engineered into such a form that is best suitable for reducing the bandgap by co-substituting different metalloids and adding graphene oxide into the ZnS lattice. The generated ZnS samples showed electronic absorption in the UV region of light, according to Fariba Soleimani et al.^28^, but the samples of ZnS in this study were modified in such a way that they display optical absorption in the visible region of light. The observed bandgap is 1.72, 1.99, 1.77, 2.01, 1.69, and 1.60 eV for the GZ, GZ:Cr, GZ:Cr-Ga, GZ:Cr-In, GZ:Cr-Al, and GO, respectively.

### Morphological study

**Figure 4:**
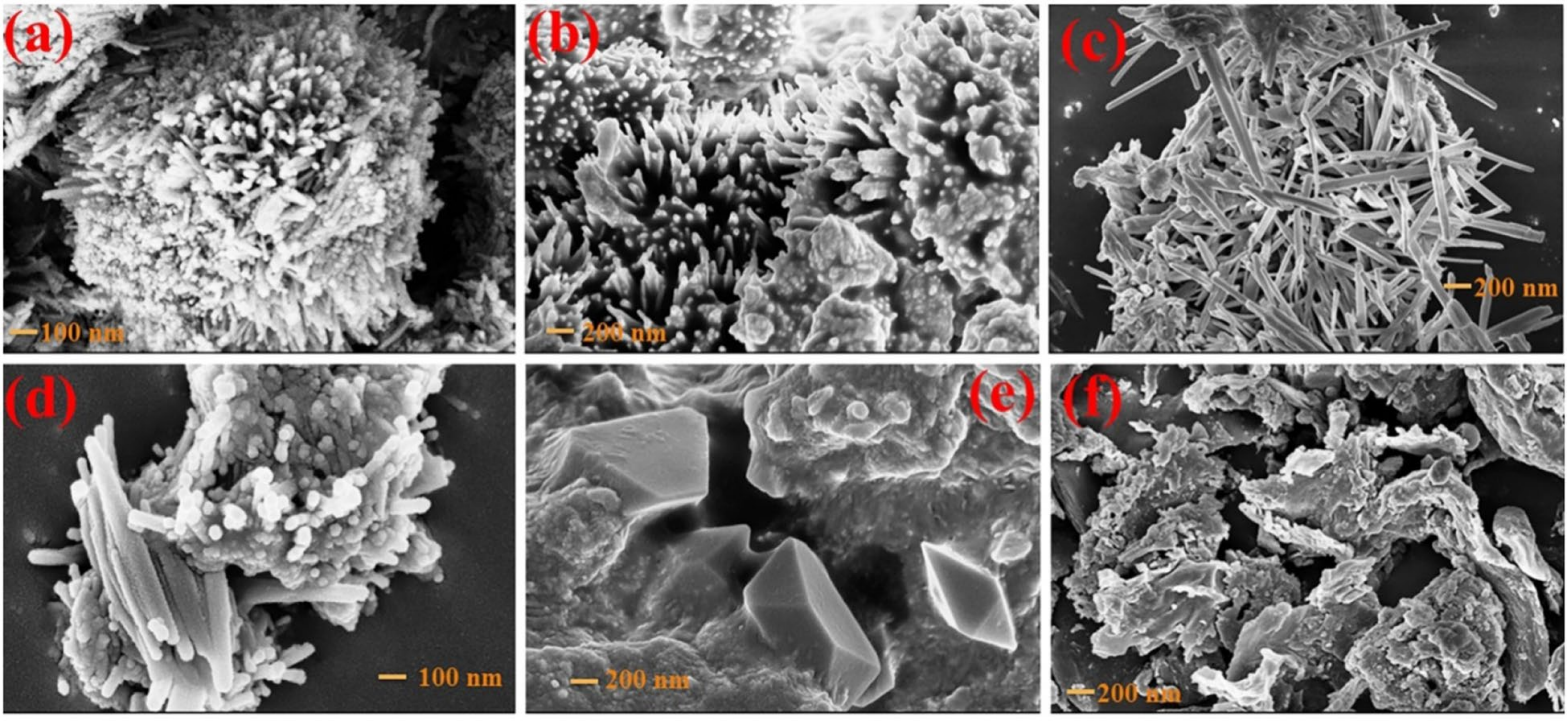
FESEM images of (**a**) GZ, (**b**) GZ:Cr, (**c**) GZ:Cr-Ga, (**d**) GZ:Cr-In, (**e**) GZ:Cr-Al, (**f**) GO samples.

Field emission scanning electron microscopy (FE-SEM) was employed to intervene morphology of the as-prepared samples. Whereas mixed morphologies offer more advantages than single crystalline structures, the current work focuses on GO-adorned co-substituted ZnS nanowires for surface modification to improve photocatalytic activities. ZnS nanocomposites have nanowire morphology in various forms, whereas GO has a nanosheet morphology which is depicted in Fig. 4. Han Zhang et al.^29^ reported that their results show that hydrothermally produced TiO_2_ microspheres with daisy, bipyramid, and sphere-like morphologies but among them, daisy microsphere morphology has exhibited the best photoexcitation performance for direct blue dye degradation, 2.3 times higher than commercial TiO_2_^30^. In samples of GZ and GZ:Cr, the nanowire forest took the form of spherical morphology. However, in GZ:Cr-Ga and GZ:Cr-In there were monocrystalline nanowires while GZ:Cr-Al shows diaphanous crystal structure morphology.

### Elemental analysis

**Figure 5:**
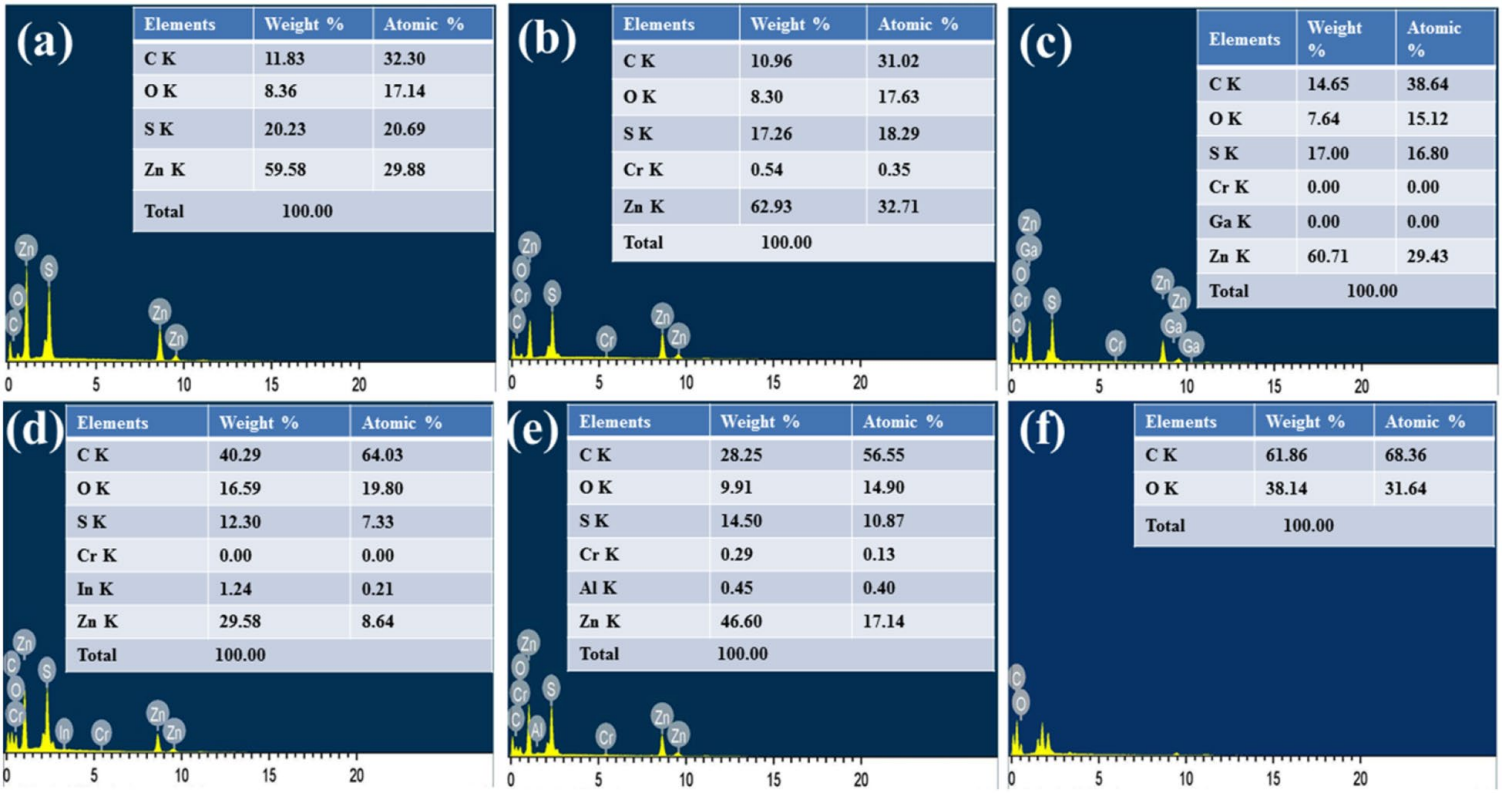
EDAX spectra of (**a**) GZ, (**b**) GZ:Cr, (**c**) GZ:Cr-Ga, (**d**) GZ:Cr-In, (**e**) GZ:Cr-Al, and (**f**) GO samples.

Chemical compositions of as-synthesized samples were determined by the EDAX technique which verifies an exact percentage of an element in the sample. It was corroborated that the existence of carbon and oxygen in samples because of the addition of GO to the ZnS lattice. The substituted metals have lower concentrations i.e. 2% of each metal but after the reaction, due to solubility, its concentration decreases, therefore, the atomic percentage of some elements shows to be 0.00%. EDAX shows the elemental atomic percentage which is equal to or greater than 2% of atomic weight. The elemental atomic percentage of respective nanocomposite samples was depicted in Fig. 5.

## Photocatalytic activity

In the last two decades, there has been enormous interest taken in photocatalytic activity, but no such development in the area of realistic dye degradation by a single photocatalyst under sunlight irradiation. Many researchers have focused on the photodegradation of one dye, its efficiency, and the reaction mechanisms. However, there are no studies to optimize several physical and chemical parameters to degrade several mixed dyes by a single photocatalyst. Therefore, we have developed a single photocatalyst that can degrade many coloured dyes. Distinct coloured dyes have been made based on colour theory. Colour theory suggests that one can form various colours primarily blue, yellow, and red. Methylene blue (blue), para nitrophenol (yellow), Methyl Orange (Orange), Triophelene (red), and Congo red (red) dyes are used for the photodegradation.

The bandgap, morphology, particle size, surface imperfections, crystallinity, surface area, thermal and chemical stability have all parameters that influence photocatalytic performance. It might be improved by optimizing bandgap, surface defects, and morphology for photogenerated electron–hole pair recombination, lowering the excitation wavelength, and increasing the number of surface-adsorbed reactant species. The development of a reduced bandgap for visible light absorption, as well as surface modification for photogenerated electron–hole pair recombination, was studied to improve photocatalytic efficiency. The azo dyes methylene blue (MB), para-nitro phenol (PNP), methyl orange (MO), congo rad (CR), and triphenylene (TP) were chosen as the mixed dye degradation to investigate the photocatalytic activity of as-prepared nanocomposite samples.

**Figure 6:**
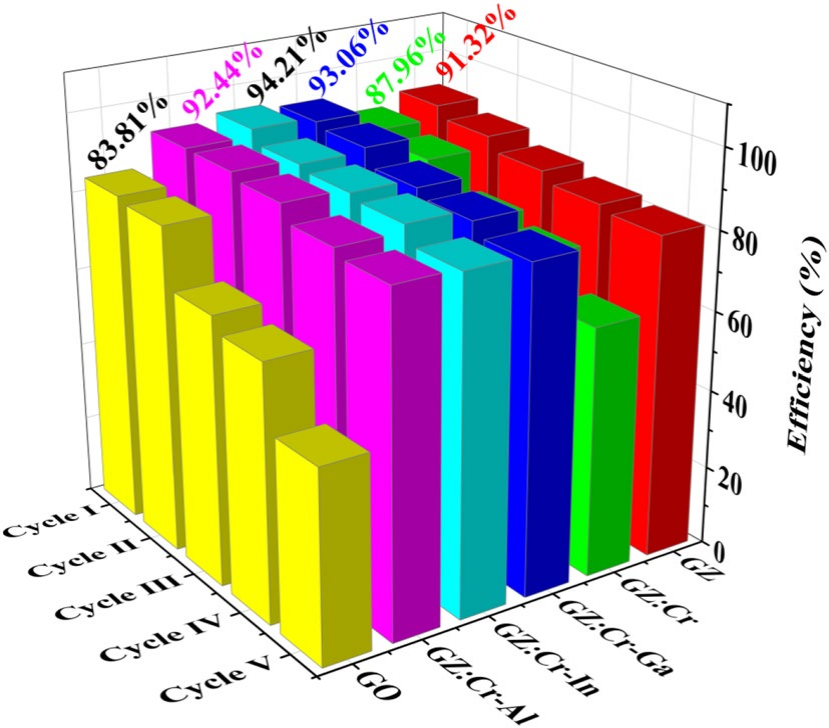
The photocatalytic efficiency (%) obtained for 5 cycles of all nanocomposites.

We have synthesized five nanocomposite samples to intervene in the photocatalytic activity of mixed dye. The photocatalytic efficiency is calculated by the following formula (9):

9 $$Photocatalytic\,efficiency \left(\%\right)=\left(\frac{{C}_{0}-{C}_{100}}{{C}_{0}}\right)\times 100$$

whereas the values of C_0_(100% dye concentration) were initial (at t = 0) and C_100_ (remaining concentration of dye after degradation) which was the final (at t = 120 min). The nanocomposites of GZ, GZ:Cr, GZ:Cr-Ga, GZ:Cr-In, GZ:Cr-Al, and GO have photocatalytic efficiencies of 91.32, 87.96, 93.06, 94.21, 92.44, and 83.81% for I cycle respectively depicted in Fig. 6. GZ:Cr-In nanocomposite exhibits the highest photocatalytic efficiency than other nanocomposites. These calculated efficiencies gave proof of co-substitution is a better option than simple semiconductor photocatalysis. After completing the five cycles, there was a deduction in photocatalytic efficiency but chemical stability after five cycles were good. In addition to efficiency, there was one more parameter that is very important in photocatalysis and i.e. rate constant which was calculated by the following formula (10):

10 $$\mathrm{log}\frac{{C}_{0}}{{C}_{t}} =kt$$

**Figure 7:**
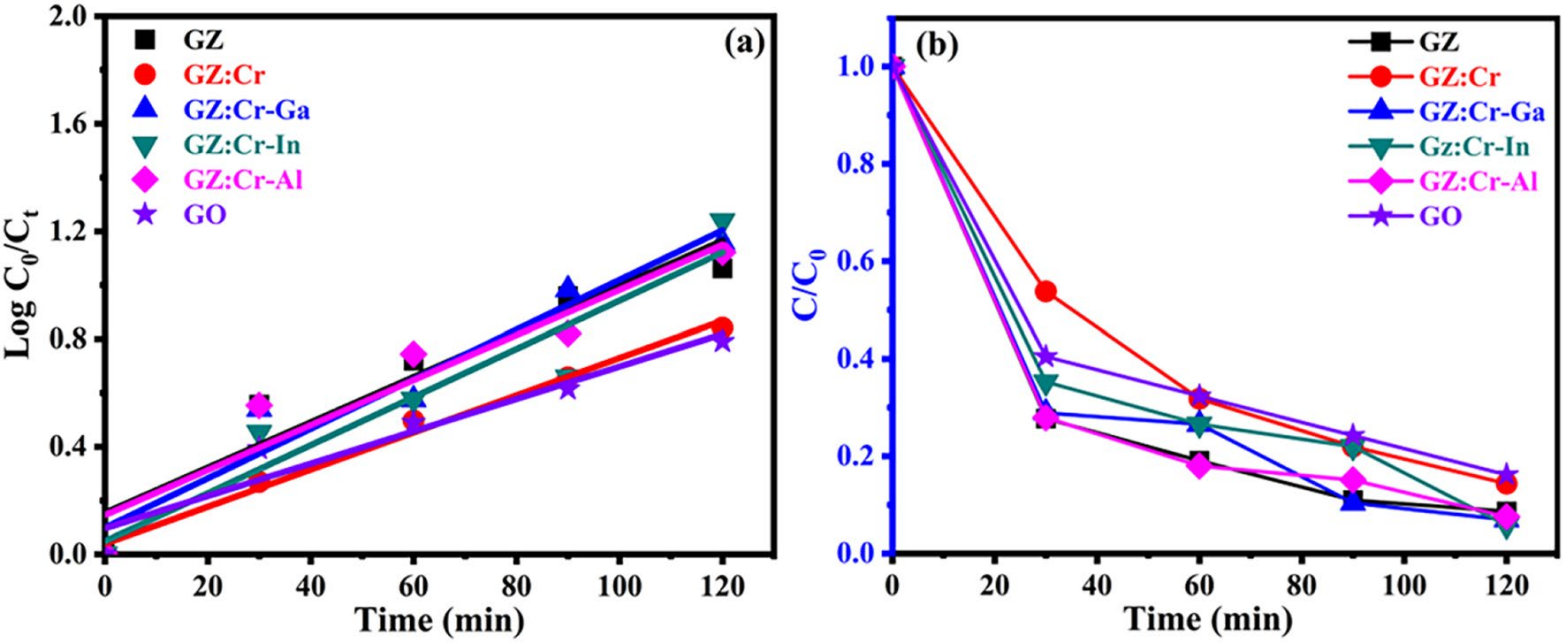
Plots of (**a**) time (min) versus Log C_0_/C_t_ and (**b**) time (min) versus C/C_0_ for the samples.

Here *C*_*0*_ at Time t = 0 min and *C*_*t*_ at time = 120 min, respectively, and k is the pseudo-first-order rate constant. The rate constant (k) was verified from the slope of $\mathrm{log}\frac{{C}_{0}}{{C}_{t}}$ versus irradiation time as depicted in Fig. 7. The verified rate constant of as-synthesized nanocomposite samples was tabulated in Table 2. From the values of photocatalytic efficiencies and rate constant, it was explicitly verified that GZ:Cr-In nanocomposite was the best photocatalyst as compared to other nanocomposite samples for degradation of mixed dyes which is depicted in Fig. 1S (ESI).

**Table 2:** Photocatalytic efficiency and pseudo rate constant of different 5 cycles.

Sample	Cycle I		Cycle II		Cycle III		Cycle IV		Cycle V	
	Photocatalytic efficiency	Pseudo rate constant	Photocatalytic efficiency	Pseudo rate constant	Photocatalytic efficiency	Pseudo rate constant	Photocatalytic efficiency	Pseudo rate constant	Photocatalytic efficiency	Pseudo rate constant
GZ	91.32	0.659	88.46	0.560	85.11	0.465	82.57	0.392	81.21	0.452
GZ:Cr	87.96	0.488	85.60	0.453	76.30	0.378	73.34	0.370	62.85	0.265
GZ:Cr-Ga	93.06	0.651	91.82	0.603	87.55	0.611	85.60	0.482	82.42	0.545
GZ:Cr-In	94.21	0.584	90.86	0.600	89.69	0.627	88.42	0.625	84.09	0.458
GZ:Cr-Al	92.44	0.648	92.30	0.665	90.69	0.646	86.66	0.539	84.85	0.445
GO	83.81	0.457	82.69	0.455	67.87	0.322	64.39	0.209	48.37	0.182

## X-ray photoelectron spectroscopy

**Figure 8:**
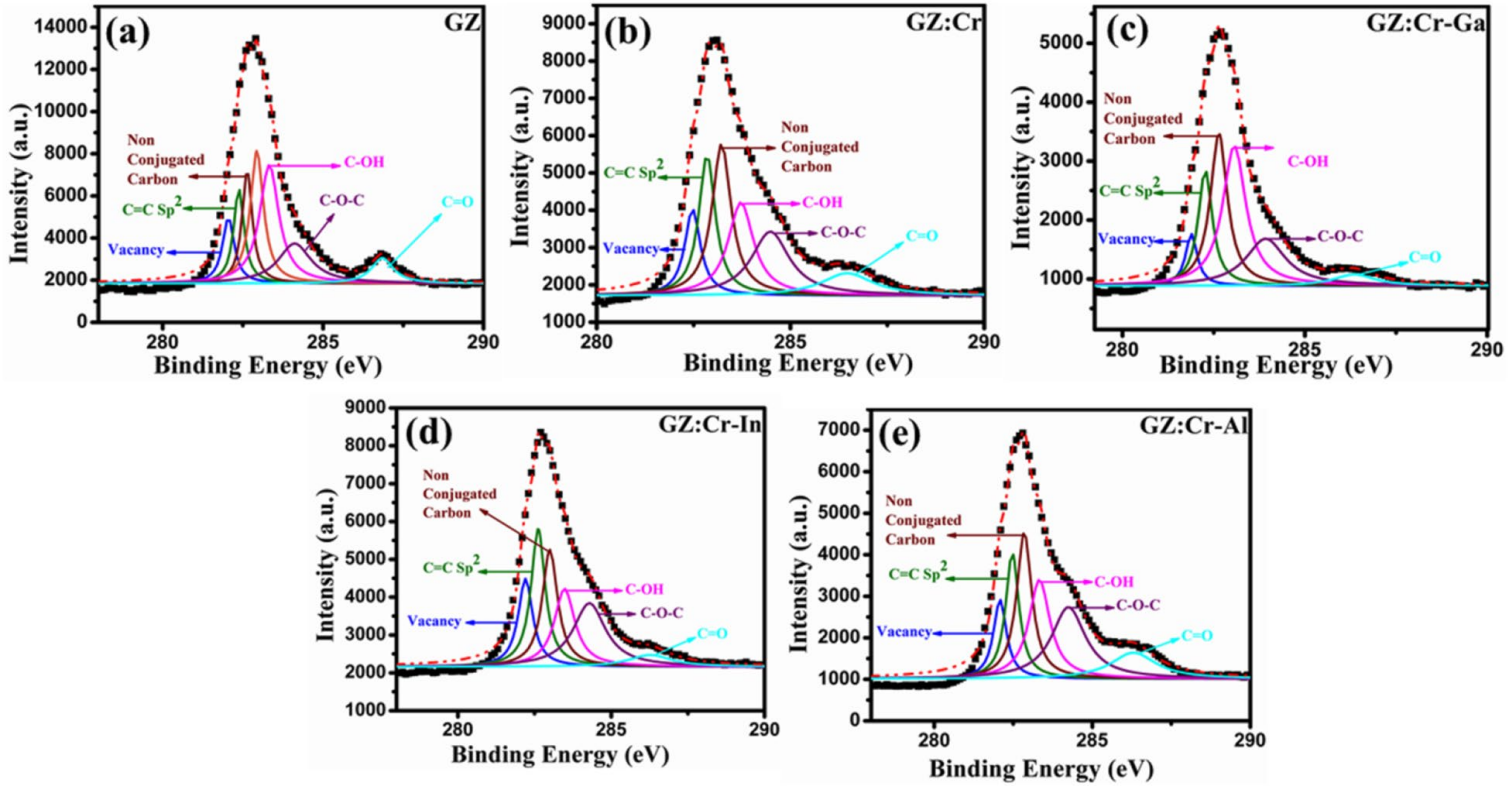
Gaussian fitted peaks of C 1 s spectra of (**a**) GZ, (**b**) GZ:Cr, (**c**) GZ:Cr-Ga, (**d**) GZ:Cr-In, and (**e**) GZ:Cr-Al.

To understand the mechanism of the photocatalytic activity in these nanocomposites, XPS was employed to extract information about surface defects, phase transformation, and the nature of chemical components present in the nanocomposites. For the determination of surface defects usually, photoluminescence spectroscopy is used but a present report has calculated surface defects from the XPS technique due to the presence of GO on the surface of ZnS nanowires. Total surface defects in percentage were calculated from non-conjugated carbon and vacancy peak^31^ which was depicted in Fig. 8. Table 3 elaborates that the area (%) under the respective curve and from that data, surface defects were analyzed. The first Gaussian fit peak represents the vacancies present in the prepared samples whereas the second Gaussian fit peak denotes the functional group C=C sp^2^. The third Gaussian peak is under the C 1 s and it represents non-conjugated carbon which also participated in the surface defect study. The fourth, fifth, and sixth Gaussian peaks represent the C–OH, C–O–C, and C=O functional groups respectively. From Table 2S (ESI)the % of elements present in prepared nanocomposite samples reveals that GZ:Cr-In nanocomposites have sulfur, nitrogen, and oxygen elements weight % was higher than other nanocomposites therefore the photocatalytic activity of GZ:Cr-In nanocomposites was higher than other samples. More oxygen vacancies were enhance the photocatalytic activity whereas a high % N doped GO also has better photocatalytic and physical properties than pristine GO. The co-substitution of 2% Ga^3+^/In^3+^/Al^3+^ along with 2% Cr^3+^ ions into ZnS lattice successfully and was verified from XPS spectra of co-substituted metals. Full scan spectra are depicted in Fig. 2S (ESI) and all metals were present as-synthesized nanocomposite samples which are depicted in Fig. 3S (ESI).

**Table 3:** Vacancy defects, non-conjugated carbon defects, C=C sp2 (area under the curve %), C–OH (area under the curve %), C–O–C (area under the curve %), C=O (area under the curve %), and total defects extracted from XPS analysis of C 1 s spectrum.

Sample	C=C sp2		NC-C		C–OH		C–O–C		C=O		Vacancy		Total defect (%)
	B.E. (eV)	Area (%)	B.E. (eV)	Area (%)	B.E. (eV)	Area (%)	B.E. (eV)	Area (%)	B.E. (eV)	Area (%)	B.E. (eV)	Area (%)	
GZ	282.37	12.87	282.93	22.91	283.32	29.72	284.1	17.18	286.84	7.25	282.04	10.03	32.94
GZ:Cr	282.47	18.44	282.84	23.18	283.22	18.90	283.71	19.42	284.48	9.21	282.47	10.81	33.26
GZ:Cr-Ga	282.26	16.38	282.64	24.01	283.06	29.42	283.92	18.72	286.35	4.65	281.88	6.06	30.07
GZ:Cr-In	282.61	21.19	283.00	19.53	283.47	17.29	284.28	22.40	286.27	5.50	282.20	13.85	33.38
GZ:Cr-Al	282.46	15.55	282.84	20.90	283.32	19.66	284.24	22.92	286.30	11.14	282.07	9.68	30.58

## Fluorescence study

**Figure 9:**
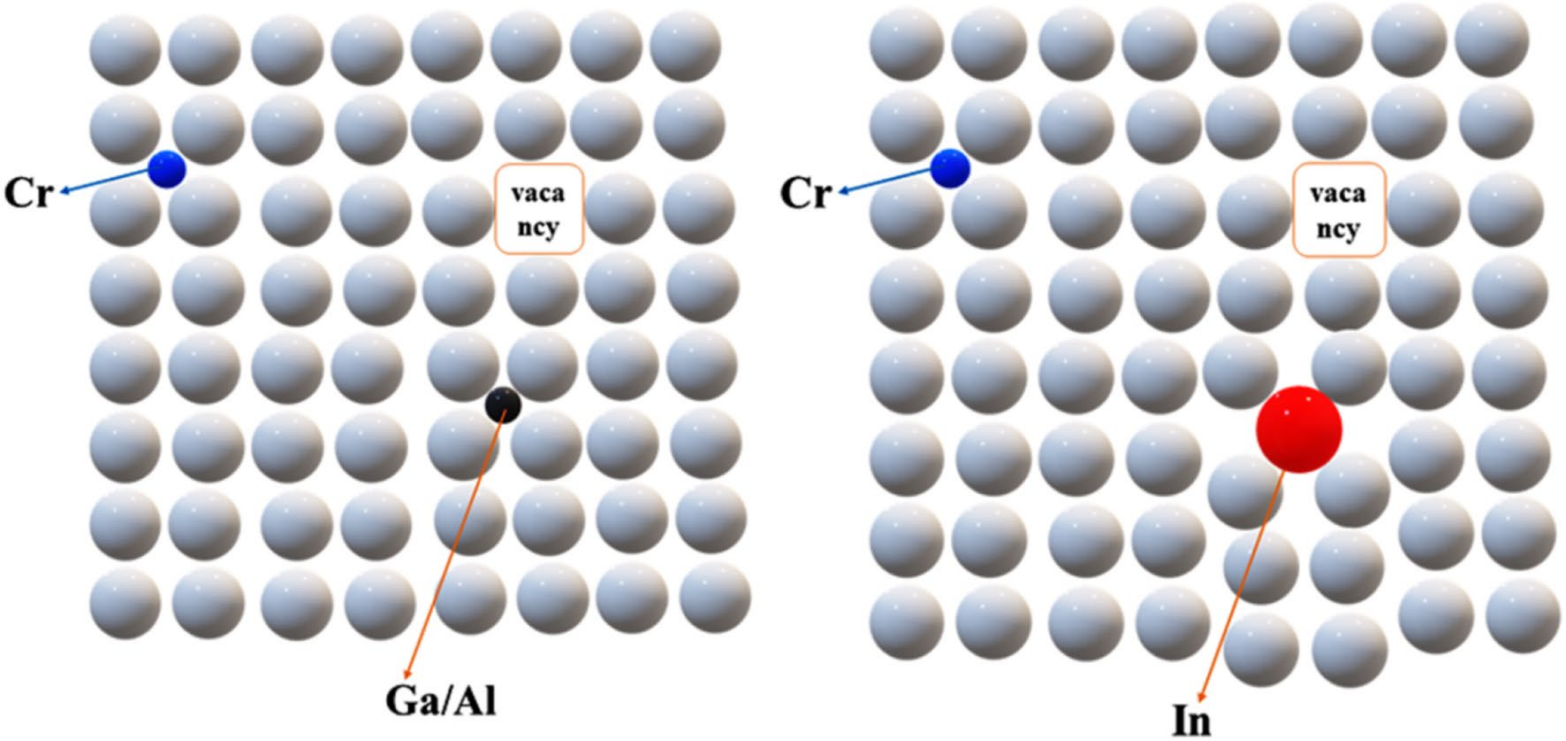
Frankel defect and substitutional defect occurred due to Cr/Ga/Al and In into ZnS lattice.

**Figure 10:**
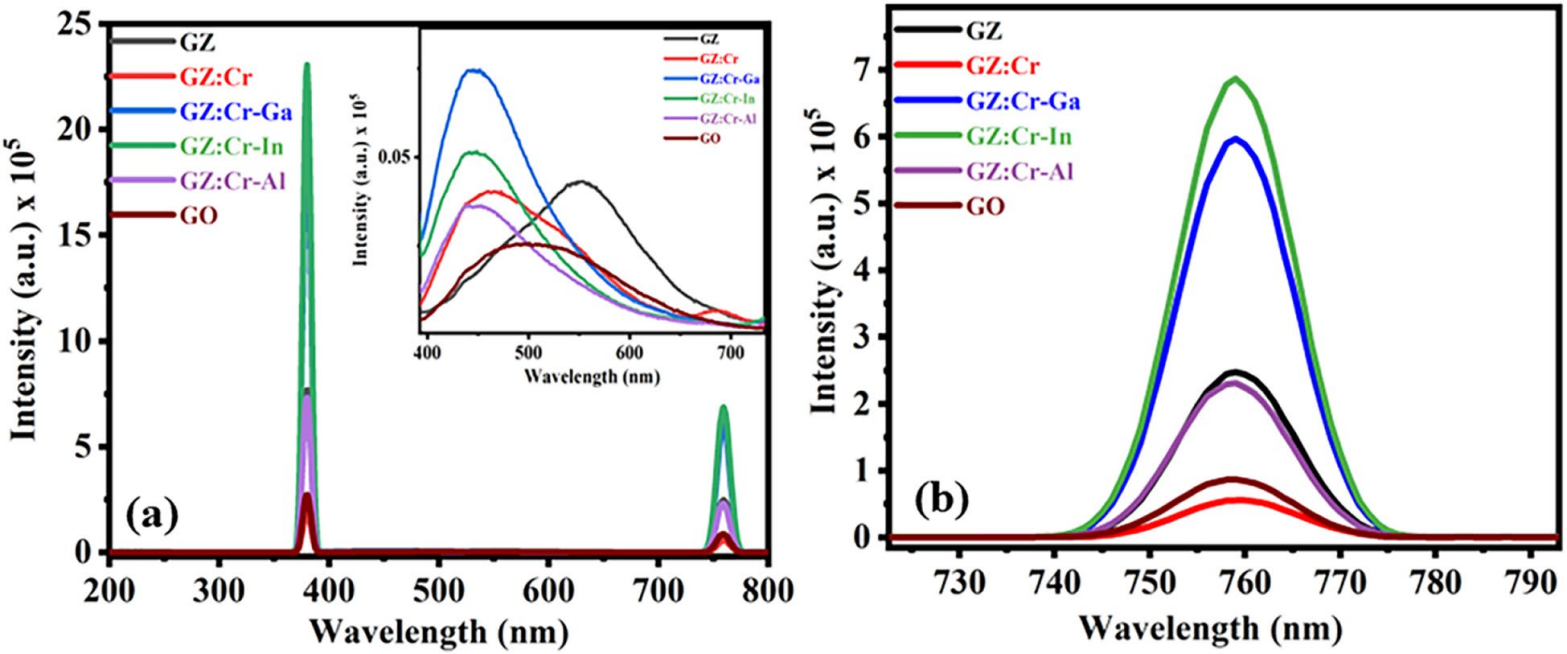
(**a**) Fluorescence spectra of prepared samples, (**b**) zoom plot in the range of 725–790 nm.

The fluorescence spectroscopy reveals the intrinsic, vacancy, substitutional, and Frankel defects which play a vital role in the enhancement of properties and photocatalytic activity of prepared samples depicted in Fig. 9. The correlation between XPS, structural characteristics (XRD), and fluorescence spectroscopy explain why synthesized samples have high photocatalytic efficiency. Due to the presence of substitutional and Frankel defects (a combination of interstitial and vacancy defects), the GZ:Cr-In has the highest photocatalytic efficiency. The sample of GZ:Cr-In has more defects in data because Cr, Ga, and Al have lower ionic radii than the host element Zn, whereas In has a larger ionic radius than Zn. From the Fluorescence spectra depicted in Fig. 10, it was observed that the strong band emission peak occurred at 380 nm whereas the defects state band peak was observed at 450 nm (violet-blue emission) and 550 nm (green-yellow emission) means it represents that a shift has occurred in prepared samples. Due to that shift, the magnetic nature of the GO and GZ was changed from diamagnetic to superparamagnetic behavior. The red emissions peak occurred at 760 nm where the greater intensity for GZ: Cr-In was due to its higher ionic radius and the formation of a large amount of substitutional and vacancy defects in its lattice site. Due to the higher intensity of defect and band emission peak, more defects were present in the GZ: Cr-In sample. From the XPS spectra, the defects were elaborated which are well matched with fluorescence and XRD data because in GZ: Cr, and GZ: Cr-Ga samples have defects due to non-conjugated carbon. From XRD these two samples have the extra peak of GO, therefore, the defects of non-conjugated carbon were formed more quantitatively here. The interstitial sulphur is mostly blamed for the strong near-band emission. The sulphur vacancy and interstitial lattice defects produced by low ionic radius elements like Cr, Ga, and Al in the ZnS nanocrystals, which are essential for photocatalytic activity, may be responsible for the violet-blue emission. The green-yellow emission is caused by self-activated defect centers linked to Zn vacancies and a larger ionic radius of In.

### Influence of morphology

The particle size and morphologies of the produced nanocomposites have an impact on photocatalytic activity. The surface area of smaller particles is higher, and this is one of the parameters that can influence photodegradation effectiveness. Three-dimensional nanospheres were developed for photocatalytic efficiency in a prior research paper, and the efficiency was found to be 84%. We first time tried to interpret the one-dimensional nanowires to enhance the photocatalytic efficiency. Nanowires were grown on the surface of spheres, and the mixed morphology outperformed the pure one in terms of photodegradation efficiency. Nanowires were adorned by GO with a smaller diameter and greater length for enhancement in porosity and surface area. Because the lengths and diameters of nanowires were diverse for distinct nanocomposite samples, therefore efficiency saturation occurred. The GO was found on the surface of nanowires, therefore improvement was observed in the active sites of nanocomposite samples.

### Impact of superparamagnetism

Researchers are studying two methods for improving photocatalytic efficiency. In the first method, they are creating an improved catalyst with outstanding photocatalytic performance so that photon consumption rates can increase. In the second method, one can alter reaction circumstances to boost photocatalytic efficiency. For example, by providing an electric field, one can drastically modify the photoinduced carrier transportation mechanism, thereby improving photocatalytic efficiency^32^. For exceptional photocatalytic performance, we have chosen the first technique with certain modifications, namely superparamagnetic N-doped GO-based co-substitution of metalloids into ZnS nanowires for higher photon consumption rates. This work will be the basis for a future connection between these approaches and methods to improve photocatalytic activity. Superparamagnetic behavior is due to thermal energy causing a fast flipping of the total magnetic moment in single-domain nanoparticles. A study suggests the nanocomposite samples were activated as superparamagnetic nanocomposites when they were exposed to sunlight during photocatalysis.

### Impact of co-substitution

This is due to the anisotropy of N-doped GO, which increases the surface area-to-volume ratio, and benefits photocatalytic activity.No reports have been published on the N-doped GO-adornedGa^3+^/In^3+^/Al^3+^ and Cr^3+^ co-substituted ZnS nanowires for Photocatalytic applications. The enhancement of photocatalytic efficiency was accomplished by co-substituting three metalloids with Cr into a ZnS lattice. The effect of Sn and Ni co-doping on a few physical properties of ZnS nanoparticles and thin films was studied by Chaitanya Kumar et al.^33^, who found that bandgap and magnetization were reduced after co-doping. Neetu Bansal et al. observed that the bandgap was enhanced after co-doping of Mn^2+^ and Cu^2+^ in the ZnS lattice^34^. Kavi Rasua et al. investigated the effect of Pd^2+^ and Mn^2+^ co-doping in the ZnS lattice which reduced the bandgap after co-coping^35^. But these reports are on cubic ZnS lattice and they have not worked for photocatalytic activity. But present work contributes to the effect of distinct metals co-substitution in ZnS lattice for photocatalytic applications. In structural parameters shifting was observed due to the diverse ionic radius of distinct metalloids. We have obtained very interesting results on co-substitution into ZnS lattice for magnetic, optical, chemical, morphological, and thermal properties also.

### Impact of defects

The photocatalytic efficiency was improved after the co-substitution of metalloids with Cr^3+^ into the ZnS lattice because interstitial, vacancy and substitutional defects were discovered in a prepared sample, providing direct evidence of photocatalytic efficiency improvement. For example, substitutional defects can introduce new active sites for photocatalytic reactions, whereas Frankel defects can increase the number of charge carriers available for the photocatalytic process when a cation vacancy is adjacent to an anion vacancy. Impurities, vacancies, and anti-site disorder can be used to improve the photocatalytic activity of ZnS by acting as recombination centers for photocarriers, resulting in an increase in charge carrier concentration and thus enhancement in photocatalytic activity. The interstitial, vacancy and substitutional defects are directly proportional to enhancement in photocatalytic efficiency, and the presence of defects was investigated from FL and XPS. Due to the existence of defects, the samples had additional charge carriers and new active sites which participate in the reaction and increase the value of the first Pseudo rate order constant and photocatalytic efficiency.

## Conclusions

Samples of nitrogen-doped GO adorned pure ZnS, 4% Cr substituted ZnS, and 2% Ga^3+^/In^3+^/Al^3+^co-substituted with 2% Cr^3+^ ions into porous GO-based ZnS nanowires were successfully prepared using a one-step hydrothermal method for Photocatalytic and biomedical applications. A drastic change in energy band gap was observed from UV–Vis reflectance spectroscopy and illustrated that the prepared nanowires are capable to absorbs more visible light. XPS and FL investigated that the presence of defects is direct evidence of the enhancement of photocatalytic efficiency. Usually, in water purification, Zn-based materials release high amounts of Zn ions with potentially toxic effects. In our case less amount of Zn reacts directly with dyes in wastewater because ZnS nanowires were decorated by the graphene sheets, therefore, the top surface is of graphene oxide which is porous and nontoxic. We outlined that the ZnS molecules are bounded between honeycomb-structured graphene oxide nanosheets therefore they are not released after the dye degradation procedure. It was verified that pristine GO is not suitable for photocatalytic activity due to its weightless property. Due to its lower weight, the recyclability of that powder is reduced therefore GO is replaced as an additive element with ZnS. There are many intriguing applications brought about by the introduction of superparamagnetic GO/ZnS and GO nanomaterials which exhibit superparamagnetic structure, including targeted drug administration, magnetic resonance imaging, magnetic hyperthermia, and thermoablation, bioseparation, and biosensing.

## Supplementary Information

Supplementary Information.

## Data Availability

All data generated or analysed during this study are included in this published article [and its supplementary information files].
