# Supplementary material for: Intriguing physicochemical properties and impact of co-dopants on N-doped graphene oxide based ZnS nanowires for photocatalytic application
Source: Sci Rep. 2023 May 10;13:7595. doi: 10.1038/s41598-023-33453-z (PMC10172323; doi:10.1038/s41598-023-33453-z)
Supplement: Supplementary file 1 — Supplementary Information. [file 41598_2023_33453_MOESM1_ESM.docx]

**Intriguing physicochemical properties and impact of co-dopants on N-doped Graphene oxide based ZnS nanowires for photocatalytic application**

*D. V. Dake^1^, N. D. Raskar^1^, V. A. Mane^1^, R. B. Sonpir^1^, E. Stathatos^2^, M. Vasundhara^3^, R. Meena^4^, K. Asokan^5^, B. N. Dole^1^**

*^1^Advanced Materials Research Laboratory, Department of Physics, Dr. Babasaheb Ambedkar Marathwada University, Aurangabad 431004, M.S., India*

*^2^Electrical and Computer Engineering Department, Nanotechnology and Advanced Materials Laboratory, University of the Peloponnese, 26334 Patras, Greece*

*^3^Polymer and Functional Department, CSIR-Indian Institution of Chemical Technology, Tarnaka, Hyderabad-500007, Telangana, India*

*^4^Materials Science, Inter-University Accelerator Centre, Aruna Asaf Ali Marg, New Delhi 110 067, India*

*^5^Department of Physics & Centre for Interdisciplinary Research, University of Petroleum and Energy Studies (UPES) Dehradun, Uttarakhand 248007 India*

**Table 1S:** Comparison between reported literature results and present work

| Sample | Method | Morphology | Dye | Degradation efficiency | Time for degradation (min) | Rate constant | Ref. No. |
| --- | --- | --- | --- | --- | --- | --- | --- |
| Ga-doped ZnS | Evaporation and condensation | Nanowires | MB | - | 10 hr | - | ^1^ |
| Ag-ZnS/rGO Nanocomposites | Ultrasound assisted co-precipitation | Nanoparticles | Tetracycline | 90.85% | 110 min | - | ^2^ |
| Fe^3+^ doped ZnS | Chemical precipitation | Quantum dots | Victoria blue R | 99.6% | 90 min | 4.55 x 10–2 /min | ^3^ |
| Cobalt-doped ZnS-RGO Nanocomposites | Co-precipitation method. | Nanoparticles | Indigo carmine | 100% | 180 min | 3.19 x 10-2 /min | ^4^ |
| S-doped-rGO/ZnS nanocomposite | Hydrothermal | Nanoparticles | 2-chlorophenol | 99.3% | 4 hr | - | ^5^ |
| La-doped ZnS | Chemical-bath deposition | Nanoparticles | Methyl orange | - | 4 hr | - | ^6^ |
| (Al, Cu) Co- doped ZnS | Chemical refluxing | Nanoparticles | Malachite green | 100% | 35 min | - | ^7^ |
| PVP-capped ZnS | Microwave irradiation method | Nanoparticles | Methylene blue | 81% | 360 min | 9.15 min^-1^ x 10^-3^ | ^8^ |
| Capped ZnS | Chemical precipitation | Nanoparticles | 2-mercaptoethanol | 92⋅6% | 180 min | 0⋅0146 s^–1^ | ^9^ |
| Strontium-doped ZnS | Hydrothermal | Nanoparticles | Methyl orange | 75% | 60 min | 10.45 × 10^−3^ min^−1^ | ^10^ |
| TiO_2_/ZnS | SILAR | Nanorods | Rhodamine B | 98% | 120 min | - | ^11^ |
| ZnS | Decomposition of zinc xanthates | Nanoparticles | Methylene blue | 60% | 180 min | - | ^12^ |
| Schiff base coordinated ZnS | Co-precipitation method | Nanoparticles | Chlorpyrifos pesticide | 85.29% | 60 min | 0.0257 min^-1^ | ^13^ |
| ZnS | A microwave-assisted solvothermal | Nanoparticles | Rhodamine B | 97% | 210 min | 14.05 x 10^-3^ min^-1^ | ^14^ |
| CdS and ZnS | Decomposition of xanthates | Nanoparticles | Methylene blue | ~61% of MB by ZnS | 180 min | 4.9 × 10^−3^ min^−1^ of ZnS | ^15^ |
| CdS-ZnS-BiPO_4_ | Solvent-assisted heating method | Nanocomposites | Methylene blue | 95% | 60 min | 0.0461 min^−1^ | ^16^ |
| CdS-ZnS | Conventional deposition process | Nanocomposites | Rifampin | 97.61% | 120 min | 0.028 min^-1^ | ^17^ |
| ZnO@ZnS core@shell Cu cable | Chemical bath deposition | Nanowires | Methylene blue, p-nitrophenol, and Rhodamine B | 98.3% ± 0.4% | 45 min | 0.13 min^-1^g^-1^ | ^18^ |
| Mn: ZnS | Chemical precipitation | Quantum Dots | Fluoroquinolone Norfloxacin | 86% | 60 min | 3.21 × 10^‒2^ min^-1^ | ^19^ |
| Zinc sulfide | Co-precipitation | Nanoparticles | Direct Blue 14 | 88.26% | 60 min | - | ^20^ |
| ZnO-ZnS core-shell nanofibers | Controlled sulfidation process | Nanofibers | Methylene blue, Rhodamine B and 4-nitrophenol | ~92% | 180 min | 0.02458 min^-1^ | ^21^ |
| CdS@ZnS Core-Shell Nanocomposites | solvothermal method | Nanowires & Nanoparticles | Methylene blue and 4-chlorophenol | 99.9% MB & 88.5% 4CP | 360 min & 720 min | - | ^22^ |
| ZnS/Ag_2_O | Hydrothermal | broccoli-like microspheres | Methylene blue | 92.4% | 50 min | 0.138 min^−1^ | ^23^ |
| C–ZnS/ZnMoO_4_@MoS_2_ | Hydrothermal | Nanosphere, Nanoflower | Tetracycline hydrochloride & Rhodamine B | 60% &100% | 120 min | 0.010 min^−1^ & 0.021 min^−1^ | ^24^ |
| CoFe2O4@ZnS core-shell nanocomposite | Hydrothermal | Nanoparticles | Methylene blue | 100% | 70 min | 0.061 min^-1^ | ^25^ |
| Hydrangea-like ZnS/ZnIn2S4 | Hydrothermal | Microspheres | Xylenol orange &Thymol blue | 100% | 40 min & 135 min | - | ^26^ |
| Au@ZnS–AgAuS Yolk–Shell Nanocrystals | Hydrothermal cation exchange synthesis | Nanocrystals | Methyl blue | - | 240 min | - | ^27^ |
| ZnS: Cu powders | - | Nanoparticles | Rhodamine B, methylene blue, and acid orange | 99.2%, 95.9%, and 78.8%, | 160 min | - | ^28^ |
| Zinc sulfide | Hydrothermal | Microspheres | Methylene blue & Rhodamine-B | 100% | 120 min | - | ^29^ |
| 2D layered MoS_2_/ZnS | Hydrothermal | Nanosheets | Methylene blue | 99.89% | 32 min | 0.1835 min^−1^ | ^30^ |
| ZnS nanoparticles@ porous Cu3SnS4 | Microwave irradiation and oil bath heating | Nanoparticles | Methyl blue | 90% | 300 min | 0.434 h^-1^ | ^31^ |
| TiO_2_/ZnS nanocomposite | Hydrothermal | Nanorods | Crystal violet | 70.07% | 60 min | 1.72 x 10^–2^ mg/cm^2^, | ^32^ |
| g-C3N4/ZnS/SnS2 ternary heterojunction | Hydrothermal | Nanospheres, Nanoparticles, Nanoplates | Methylene blue | 95% | 20 min | 0.148 min^-1^ | ^33^ |
| Copper assisted ZnS | SILAR method | Nanoparticles | Methylene blue | 56% | 360 min | - | ^34^ |
| ZnS quantum dots- β-Bi2O4 Nanosheets Nanocomposites | Precipitation, solvothermal, and wet impregnation route | quantum dots & Nanosheets | Methylene blue & Rhodamine-B | 93.77% & 94.34% | 120 min & 180 min | (0.019 & 0.016) min^-1^ | ^35^ |
| Pristine ZnS and Cr doped ZnS | Hydrothermal | Nanosheet based nanospheres formed from nanocubes and pure nanocubes | MB, MO, mixed dye | 74.67%, 80.01%, 75.10% | 100 min, 140 min, 240 min | 0.0034, 0.00376, 0.00249 min-1 | ^36^ |
| Graphene-based Cr substituted β ZnS | Hydrothermal | Nanospheres | MO/MB/CR mixed dye | 84.49% | 180 min | 0.3595 min-1 | ^37^ |
| 2% Ga^3+^/In^3+^/Al^3+^ and 2% Cr^3+^ co-substituted ZnS/N doped GO nanocomposites | Hydrothermal | Nanowires | Mixed dye | 94.21% | 120 min | 0.5848min^-1^ | [Present work] |

**Experimental**

**Material characterization**

The prepared nanocomposite samples were characterized by diverse techniques such as XRD, XPS, VSM, FESEM with EDS, and UV-Vis spectroscopy. Structural properties such as lattice parameters, crystallite size, volume, dislocation density, microstrain, stacking faults, ‘U’ parameter, bond length, and c/a ratio were intervened by using the XRD technique (D-8 Advanced diffractometer, Bruker AXS, Germany). Surface defects and qualitative and quantitative chemical analysis were investigated by using X-ray photoelectron spectroscopy. Enhancement in the magnetic behavior of prepared samples was scrutinized by using a vibrating sample magnetometer. The mixed morphology of the synthesized samples was tested by using field emission scanning electron microscopy. Elemental analysis was validated by energy dispersive spectroscopy. Semiconductor properties such as the energy bandgap were illustrated using UV-Visible spectroscopy.

**Photocatalytic procedure**

Dye degradation is one of the photocatalytic applications that was investigated by estimating the proportion of dye removed from decontaminated water when exposed to solar light. For the photocatalytic experiment, 100 ml water, 20 mg/L dye powder (each), and 0.05 g photocatalyst powder were employed. In the darkroom, 20 mg/L of each dye powder was soaked in 100 ml of water and stirred for 10 minutes. Following the preparation of the dye aqueous solution, 0.05 g of photocatalyst powder was scattered into the dye solution and stirred for 20 minutes in the darkroom to avoid sunlight irradiation. After completing the stirring for an aqueous solution, these samples were degraded during the daytime from 11 a.m. to 1 p.m. under solar irradiation in Aurangabad, Maharashtra, India. The latitude and longitude are respectively 19.850 N and 75.350 E. The average daily solar intensity was determined to be between 3.0 and 7.5 kWh/m^2^/day. About 3 ml of the solution was introverted at 20-minute intervals of illumination, and the UV-Vis spectra of the degraded solution were recorded to monitor from the recorded absorption peak to determine the level of degradation of each species. The photocatalytic breakdown of the dyes is depicted in Scheme 1 as a series of phases. During this procedure, photocatalyst powder dropped to the bottom of the beaker and was collected for use in recycling degradation. We have developed a single photocatalyst that degrades many coloured dyes. Distinct coloured dyes have been made based on colour theory. Colour theory suggests that many colours will be formed from the basic three colours, such as blue, yellow, and red, which are the primary colours. Methylene blue (blue), para nitrophenol (yellow), Methyl Orange (Orange), Triophelene (red), and Congo red (red) dyes were used for the photodegradation. After the degradation of dyes, the photocatalyst was recovered by centrifugation at 15000 rpm and used for the next cycle.

**Photocatalytic Activity:**


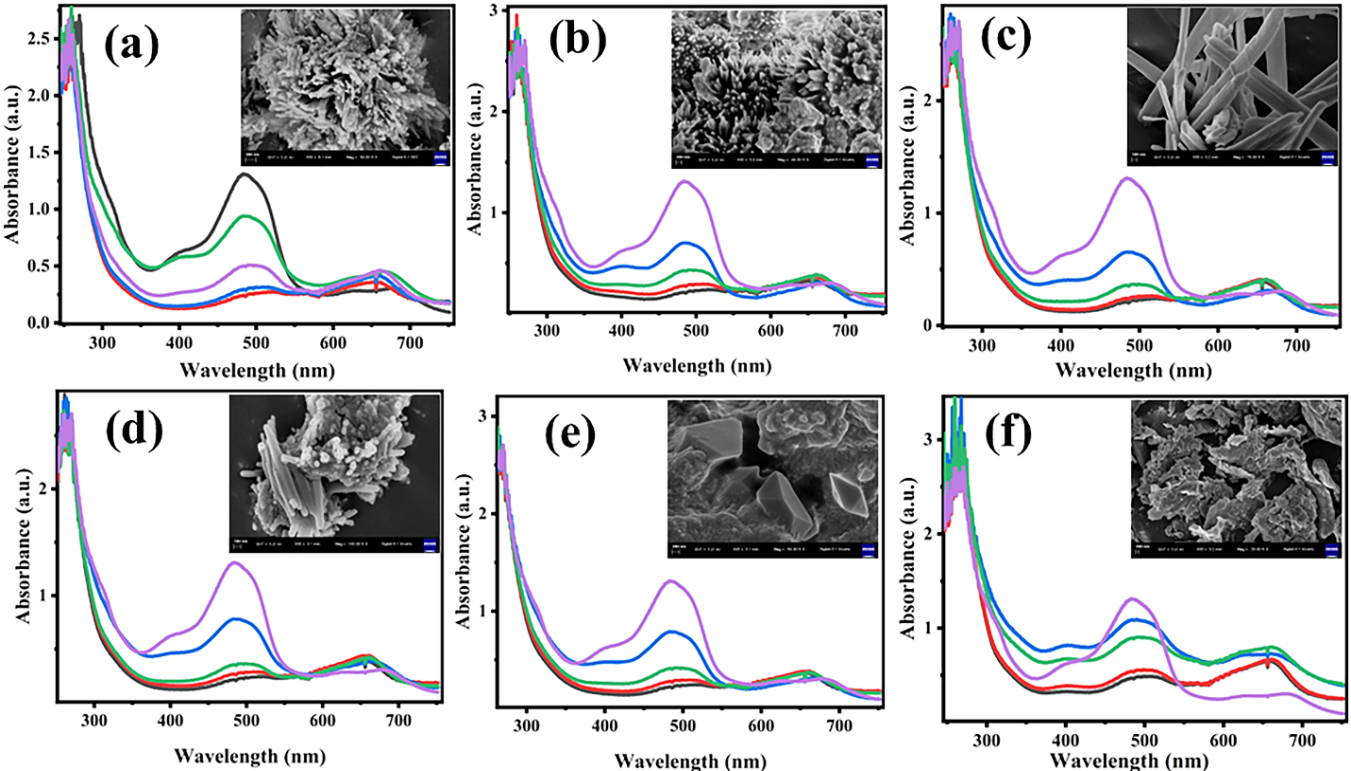


**Fig. 1S.** Photodegradation of methylene blue (MB), para-nitro phenol (PNP), methyl orange (MO), congo rad (CR), and triophelene (TP) mixed dye by using the (a) GZ, (b) GZ:Cr, (c) GZ:Cr-Ga, (d) GZ:Cr-In, and (e) GZ:Cr-Al, (f) GO

**X-ray Photoelectron Spectroscopy (XPS):**

**Table 2S:** Atomic percentage of the respective elements exist in synthesized nanocomposite samples

| **Sample** | **Zn** | **Cr** | **S** | **C** | **N** | **O** | **Other co-dopant with Cr** |
| --- | --- | --- | --- | --- | --- | --- | --- |
| **GZ** | 53.556 | 0.00 | 9.158 | 31.769 | 0.735 | 4.780 | 0.00 |
| **GZ:Cr** | 41.135 | 0.840 | 16.03 | 27.077 | 9.719 | 5.188 | 0.00 |
| **GZ:Cr-Ga** | 36.764 | 0.947 | 14.197 | 33.061 | 5.701 | 8.765 | **Ga:** 0.557 |
| **GZ:Cr-In** | 47.189 | 1.215 | 16.673 | 24.013 | 5.883 | 3.542 | **In:** 1.476 |
| **GZ:Cr-Al** | 32.7 | 2.28 | 10.35 | 37.25 | 10.31 | 6.87 | **Al:** 0.24 |


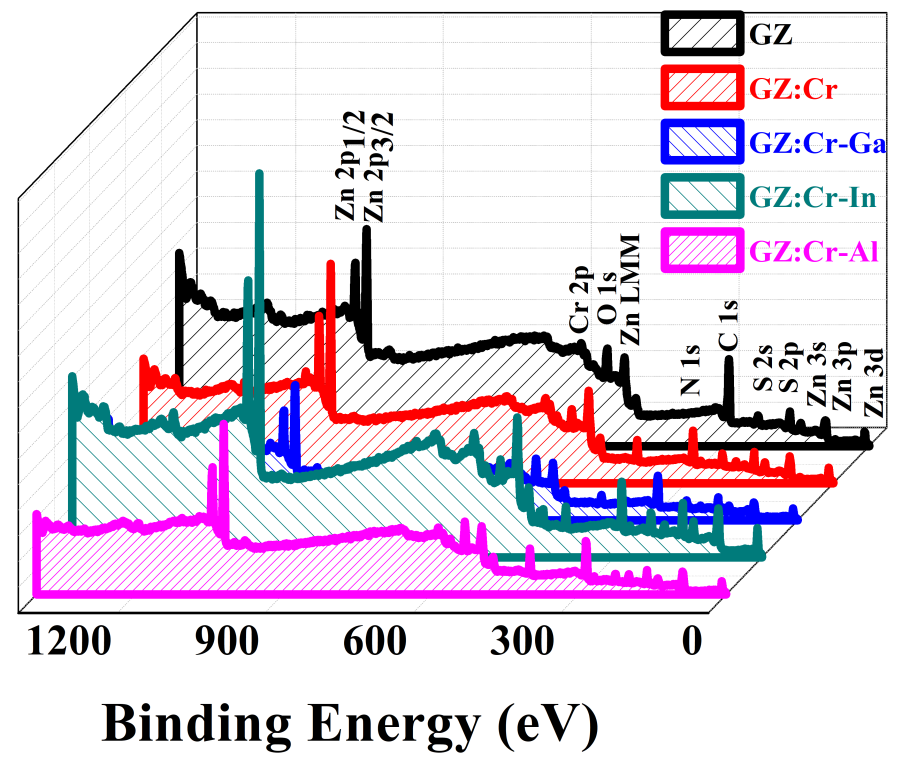


**Fig. 2S.** Full scan of the samples, high-resolution XPS spectra of the nanocomposite samples


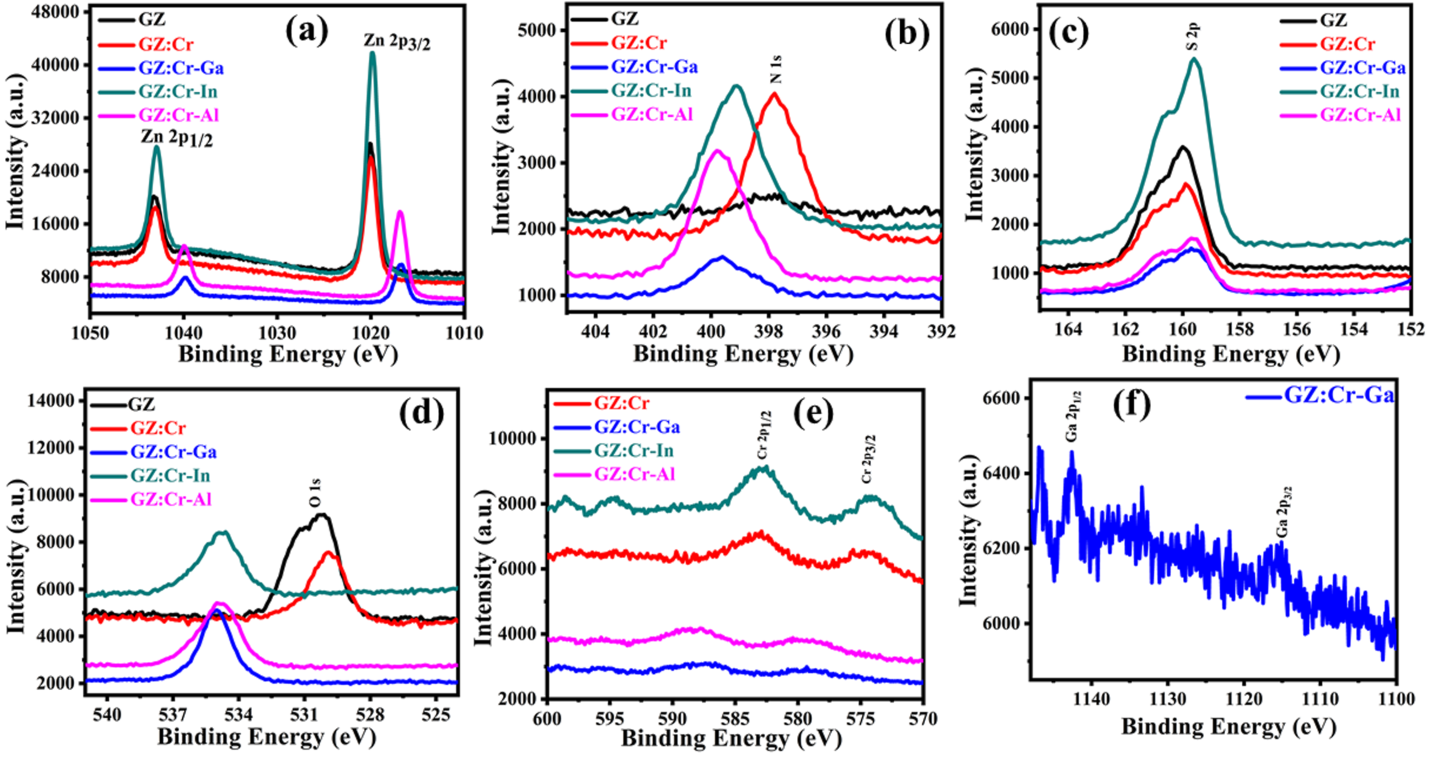


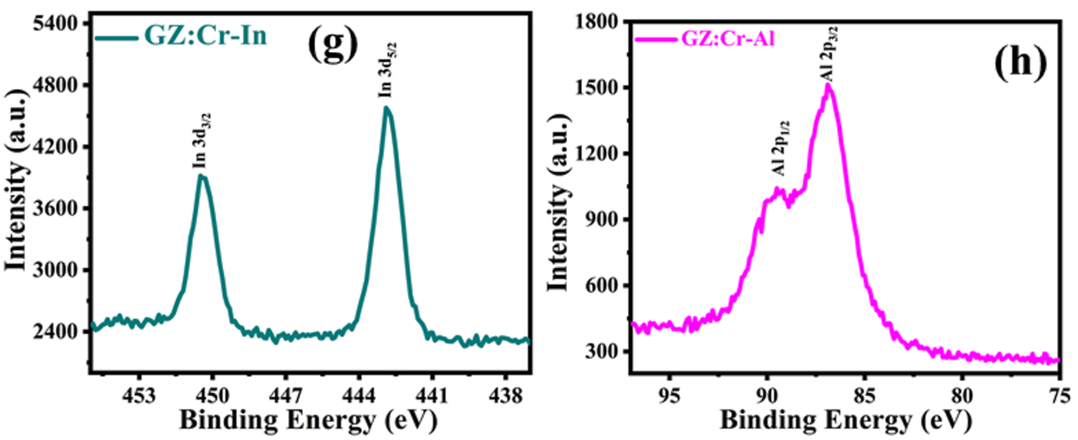


**Fig. 3S.** XPS spectra of (a) Zn 2p, (b) N 1s, (c) S 2p, (d) O 1s, (e) Cr 2p, (f) Ga 2p, (g) In 3d, (h) Al 2p

1. **Magnetic investigations: Vibrating Sample Magnetometer (VSM):**

At 300 K, varying magnetic fields between -90 kOe and +90 kOe were applied to record magnetization loops (M- H) of as-prepared nanocomposite samples using the VSM technique. All hysteresis loops were demonstrated in Fig. 4S. Nanocomposite samples of GZ:Cr, GZ:Cr-Ga, GZ:Cr-In, and GZ:Cr-Al are diamagnetic. Although the samples of GZ, and GO have a shape that resembles the letter 'S,' indicating ferromagnetic behavior, the samples of GZ, and GO have no saturation magnetization, indicating that nanocomposite samples are superparamagnetic. The magnetic property of the as-prepared nanocomposite samples is directly proportional to Zn, and C vacancies whereas inversely proportional to S vacancies. The phenomenon is called superparamagnetic found in nanoparticles which is a form of magnetism formed in ferromagnetic or ferrimagnetic nanomaterials. Nanoparticles in the absence of an external magnetic field appear to have zero magnetization when measured over a period of time longer than the Neels relaxation time, which is known as the superparamagnetic state ^38^. It is possible to magnetize the nanoparticles in this state, similar to a paramagnet, but their magnetic susceptibility is much greater than that of a paramagnet. Generally, as-prepared nanocomposite samples such as GZ, and GO have superparamagnetic structures occurred due to their single magnetic domain, which is likely to have a diameter of nanowires less than 3–50 nm. Single magnetic domain was verified based on the squareness ratio of samples exhibited less than or equal to 0.5 that samples have single domain structure whereas greater than 0.5 then it has multi-domain structure. The magnetic parameters such as magnetic saturation [Ms (emu/g)], remanence magnetization [Mr (emu/g)], coercivity [Hc (Oe)], Squareness ratio, and Bohr magnetron number of as-prepared nanocomposite samples were summarized in Table 3S. There are many intriguing applications brought about by the introduction of superparamagnetic nanoparticles which exhibit superparamagnetic structure, including targeted drug administration, magnetic resonance imaging, magnetic hyperthermia and thermoablation, bioseparation, and biosensing ^39^.


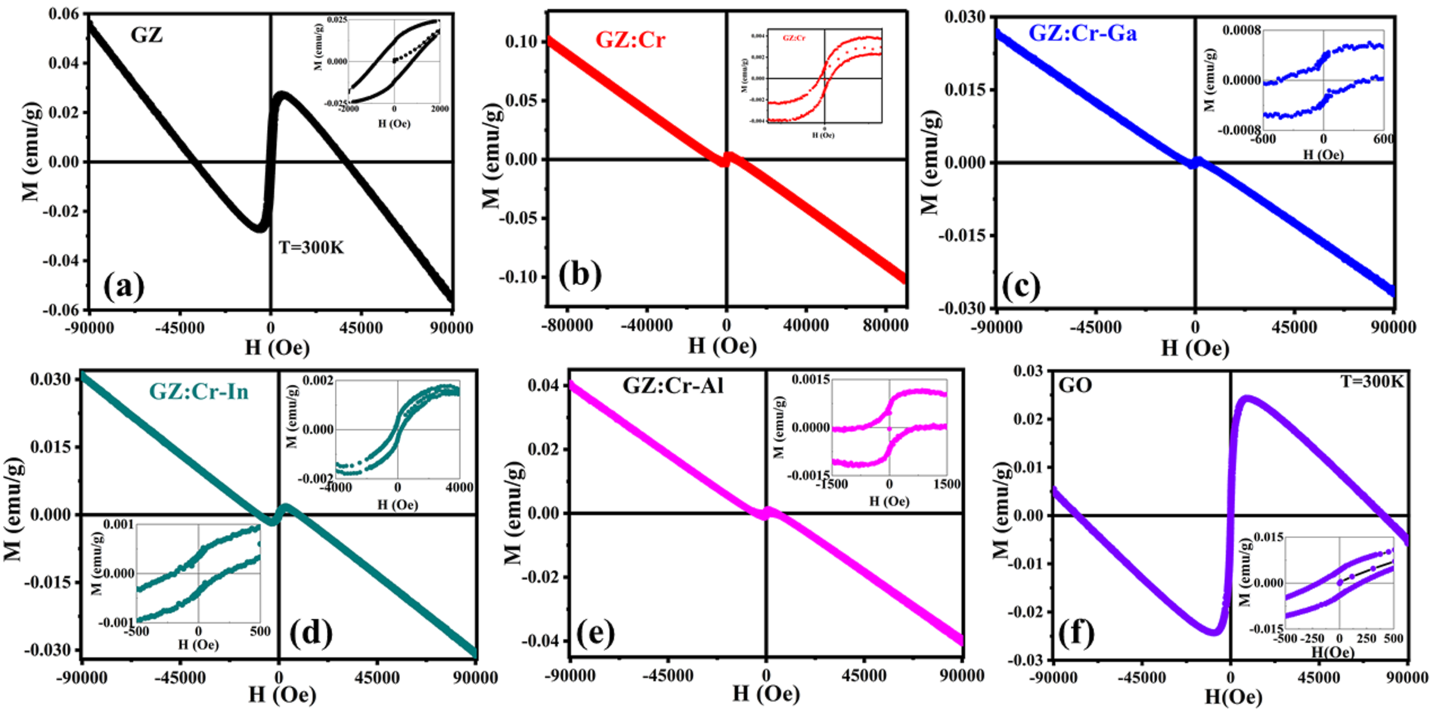


**Fig.4S** Hysteresis curves of (a) GZ, (b) GZ:Cr, (c) GZ:Cr-Ga, (d) GZ:Cr-In, (e) GZ:Cr-Al, and (f) GO samples.

**Table 3S:** The vibrating sample magnetometer data such as magnetic saturation [Ms (emu/g)], remanence magnetization [Mr (emu/g)], coercivity [Hc (Oe)], Squareness ratio, and Bohr magnetron number of prepared nanocomposite samples.

| **Sample** | **M_s_ (emu/g)** | **M_r_ (emu/g)** | **H_c_ (Oe)** | **Squareness** | **Bohr Magnetron Number** |
| --- | --- | --- | --- | --- | --- |
| GZ | 0.027 | 0.012 | 774.35 | 0.445 | 5.484610^-4^ |
| GZ:Cr | 0.002 | 0.001 | 155.11 | 0.5 | 4.137110^-5^ |
| GZ:Cr-Ga | 0.0003 | 0.0003 | 627 | 1.00 | 6.224510^-6^ |
| GZ:Cr-In | 0.001 | 0.0004 | 274.52 | 0.4 | 2.091110^-5^ |
| GZ:Cr-Al | 0.0006 | 0.0006 | 1881 | 1.00 | 1.235710^-5^ |
| **GO** | 0.024 | 0.005 | 205 | 0.208 | 6.871210^-5^ |

**References:**

1. Chen Y-C, Wang C-H, Lin H-Y, Li B-H, Chen W-T, Liu C-P. Growth of Ga-doped ZnS nanowires constructed by self-assembled hexagonal platelets with excellent photocatalytic properties. *Nanotechnology* 2010, **21**(45)**:** 455604.

2. Kameli S, Mehrizad A. Ultrasound-assisted Synthesis of Ag-ZnS/rGO and its Utilization in Photocatalytic Degradation of Tetracycline Under Visible Light Irradiation. *Photochemistry and Photobiology* 2019, **95**(2)**:** 512-521.

3. Shamsipur M, Reza Rajabi H, Khani O. Pure and Fe3+-doped ZnS quantum dots as novel and efficient nanophotocatalysts: Synthesis, characterization and use for decolorization of Victoria blue R. *Materials Science in Semiconductor Processing* 2013, **16**(4)**:** 1154-1161.

4. Agorku ES, Mamo MA, Mamba BB, Pandey AC, Mishra AK. Cobalt-doped ZnS-reduced graphene oxide nanocomposite as an advanced photocatalytic material. *Journal of Porous Materials* 2015, **22**(1)**:** 47-56.

5. Alafif ZO, Anjum M, Ansari MO, Kumar R, Rashid J, Madkour M*, et al.* Synthesis and characterization of S-doped-rGO/ZnS nanocomposite for the photocatalytic degradation of 2-chlorophenol and disinfection of real dairy wastewater. *Journal of Photochemistry and Photobiology A: Chemistry* 2019, **377:** 190-197.

6. Chen Y, Huang G-F, Huang W-Q, Zou BS, Pan A. Enhanced visible-light photoactivity of La-doped ZnS thin films. *Applied Physics A* 2012, **108**(4)**:** 895-900.

7. Poornaprakash B, Chalapathi U, Poojitha PT, Vattikuti SVP, Reddy MSP. (Al, Cu) Co-doped ZnS nanoparticles: structural, chemical, optical, and photocatalytic properties. *Journal of Materials Science: Materials in Electronics* 2019, **30**(10)**:** 9897-9902.

8. Soltani N, Saion E, Mahmood Mat Yunus W, Navasery M, Bahmanrokh G, Erfani M*, et al.* Photocatalytic degradation of methylene blue under visible light using PVP-capped ZnS and CdS nanoparticles. *Solar Energy* 2013, **97:** 147-154.

9. Kaur J, Sharma M, Pandey OP. Synthesis, characterization, photocatalytic and reusability studies of capped ZnS nanoparticles. *Bulletin of Materials Science* 2014, **37**(4)**:** 931-940.

10. Boulkroune R, Sebais M, Messai Y, Bourzami R, Schmutz M, Blanck C*, et al.* Hydrothermal synthesis of strontium-doped ZnS nanoparticles: structural, electronic and photocatalytic investigations. *Bulletin of Materials Science* 2019, **42**(5)**:** 223.

11. Li X, Shi Z, Liu J, Wang J. Shell thickness dependent photocatalytic activity of TiO2/ZnS core-shell nanorod arrays. *Materials Research Express* 2020, **6**(12)**:** 1250b1253.

12. Mintcheva N, Gicheva G, Panayotova M, Kulinich SA. Room-Temperature Synthesis of ZnS Nanoparticles Using Zinc Xanthates as Molecular Precursors. *Materials* 2020, **13**(1)**:** 171.

13. Ayodhya D, Veerabhadram G. Fabrication of Schiff base coordinated ZnS nanoparticles for enhanced photocatalytic degradation of chlorpyrifos pesticide and detection of heavy metal ions. *Journal of Materiomics* 2019, **5**(3)**:** 446-454.

14. La Porta FA, Nogueira AE, Gracia L, Pereira WS, Botelho G, Mulinari TA*, et al.* An experimental and theoretical investigation on the optical and photocatalytic properties of ZnS nanoparticles. *Journal of Physics and Chemistry of Solids* 2017, **103:** 179-189.

15. Mintcheva N, Gicheva G, Panayotova M, Wunderlich W, Kuchmizhak AA, Kulinich SA. Preparation and Photocatalytic Properties of CdS and ZnS Nanomaterials Derived from Metal Xanthate. *Materials* 2019, **12**(20)**:** 3313.

16. Tsai H-C, Peng Y-H, Wen P-Y, Wu T, Lin Y-W. Enhanced Visible Light Photocatalytic Degradation of Methylene Blue by CdS-ZnS-BiPO4 Nanocomposites Prepared by a Solvent-Assisted Heating Method. *Catalysts* 2021, **11**(9)**:** 1095.

17. Soleimani F, Nezamzadeh-Ejhieh A. Study of the photocatalytic activity of CdS–ZnS nano-composite in the photodegradation of rifampin in aqueous solution. *Journal of Materials Research and Technology* 2020, **9**(6)**:** 16237-16251.

18. Serrà A, Philippe L. Simple and scalable fabrication of hairy ZnO@ZnS core@shell Cu cables for continuous sunlight-driven photocatalytic water remediation. *Chemical Engineering Journal* 2020, **401:** 126164.

19. Patel J, Singh AK, Carabineiro SAC. Assessing the Photocatalytic Degradation of Fluoroquinolone Norfloxacin by Mn:ZnS Quantum Dots: Kinetic Study, Degradation Pathway and Influencing Factors. *Nanomaterials* 2020, **10**(5)**:** 964.

20. Mehrizad A, Gharbani P. Optimization of operational variables and kinetic modeling for photocatalytic removal of Direct Blue 14 from aqueous media by ZnS nanoparticles. *Journal of Water and Health* 2017, **15**(6)**:** 955-965.

21. Ranjith KS, Senthamizhan A, Balusamy B, Uyar T. Nanograined surface shell wall controlled ZnO–ZnS core–shell nanofibers and their shell wall thickness dependent visible photocatalytic properties. *Catalysis Science & Technology* 2017, **7**(5)**:** 1167-1180.

22. Wang L, Wei H, Fan Y, Liu X, Zhan J. Synthesis, Optical Properties, and Photocatalytic Activity of One-Dimensional CdS@ZnS Core-Shell Nanocomposites. *Nanoscale Research Letters* 2009, **4**(6)**:** 558.

23. Yu D, Fang H, Qiu P, Meng F, Liu H, Wang S*, et al.* Improving the Performance of ZnS Photocatalyst in Degrading Organic Pollutants by Constructing Composites with Ag2O. *Nanomaterials* 2021, **11**(6)**:** 1451.

24. Cui Y-W, Zhang H-H, Yu S-Y. Constructing ZIF-8 derived C–ZnS/ZnMoO4@MoS2 and C–ZnS/MoS2 nanocomposites using a simple one-pot strategy to enhance photocatalytic degradation activity. *RSC Advances* 2019, **9**(60)**:** 35189-35196.

25. Farhadi S, Siadatnasab F, Khataee A. Ultrasound-assisted degradation of organic dyes over magnetic CoFe2O4@ZnS core-shell nanocomposite. *Ultrasonics Sonochemistry* 2017, **37:** 298-309.

26. Fa D, Miao Y. Hydrangea-like ZnS/ZnIn2S4 microspheres with outstanding photocatalytic degradation of xylenol orange and thymol blue under vis irradiation. *Micro & Nano Letters* 2021, **16**(10)**:** 500-505.

27. Feng J, Liu J, Cheng X, Liu J, Xu M, Zhang J. Hydrothermal Cation Exchange Enabled Gradual Evolution of Au@ZnS–AgAuS Yolk–Shell Nanocrystals and Their Visible Light Photocatalytic Applications. *Advanced Science* 2018, **5**(1)**:** 1700376.

28. Luo W, Ying J, Yu S, Yang X, Jia Y, Chen M*, et al.* ZnS:Cu powders with strong visible-light photocatalysis and pyro-catalysis for room-temperature dye decomposition. *Ceramics International* 2020, **46**(8, Part B)**:** 12096-12101.

29. Zhang J, Yang L, Cui Y, Tang YL, Xu JY, Su ZC*, et al.* The role of luminescence self-absorption in photocatalytic properties of self-assembled ZnS nanocrystals. *AIP Advances* 2020, **10**(10)**:** 105314.

30. Harish S, Prachi, Archana J, Navaneethan M, Shimomura M, Ikeda H*, et al.* Synergistic interaction of 2D layered MoS2/ZnS nanocomposite for highly efficient photocatalytic activity under visible light irradiation. *Applied Surface Science* 2019, **488:** 36-45.

31. Shen T, Liu G, Wei L, Zhu Y, Sun S. Construction of ZnS nanoparticles@ porous Cu3SnS4 P-N heterojunction for simulated natural sunlight degradation of methyl blue. *Materials Letters* 2019, **253:** 446-449.

32. Priya GH, Shaly AA, Linet JM. Effect of Zn variation in TiO2/ZnS nanocomposite on photocatalysis for the degradation of the hazardous crystal violet dye. *Journal of Materials Science: Materials in Electronics* 2021, **32**(5)**:** 5790-5802.

33. Dai K, Lv J, Zhang J, Liang C, Zhu G. Band structure engineering design of g-C3N4/ZnS/SnS2 ternary heterojunction visible-light photocatalyst with ZnS as electron transport buffer material. *Journal of Alloys and Compounds* 2019, **778:** 215-223.

34. Mehrabian M, Esteki Z. Degradation of methylene blue by photocatalysis of copper assisted ZnS nanoparticle thin films. *Optik* 2017, **130:** 1168-1172.

35. G P, K B, T P, R S, Katubi KM, Alsaiari NS*, et al.* ZnS quantum dots and Bi metals embedded with two dimensional β-Bi2O4 nanosheets for efficient UV-visible light driven photocatalysis. *Materials Research Bulletin* 2021, **142:** 111387.

36. Dake DV, Raskar ND, Mane VA, Sonpir RB, Stathatos E, Asokan K*, et al.* Exploring the role of defects on diverse properties of Cr-substituted ZnS nanostructures for photocatalytic applications. *Applied Physics A* 2020, **126**(8)**:** 640.

37. Dake DV, Raskar ND, Mane VA, Sonpir RB, Khawal HA, Deshpande U*, et al.* Photocatalytic performance of graphene-based Cr-substituted β ZnS nanocomposites. *Applied Physics A* 2022, **128**(4)**:** 276.

38. Binns C. Tutorial Section on Nanomagnetism. *Frontiers of Nanoscience* 2014, **6:** 1-32.

39. Montazer M, Harifi T. Magnetic nanofinishes for textiles. 2018, pp 225-240.
